# Integrated microfluidic platforms for extracellular vesicles: Separation, detection, and clinical translation

**DOI:** 10.1063/5.0273892

**Published:** 2025-09-09

**Authors:** Yang Dai, Yibo Cui, Jinwen Li, Piwu Li, Xiaowen Huang

**Affiliations:** 1 State Key Laboratory of Biobased Material and Green Papermaking, College of Bioengineering, Qilu University of Technology (Shandong Academy of Sciences), Jinan, Shandong 250353, China; 2 Institute of Brain Science and Brain-Inspired Research, Shandong First Medical University & Shandong Academy of Medical Sciences, Jinan, Shandong 250000, China

## Abstract

Extracellular vesicles (EVs), secreted by most living cells, encapsulate a diverse array of bioactive molecules from their parent cells, including proteins and nucleic acids. Recent studies underscore the potential of EVs as advanced biomarkers for the early diagnosis of a variety of clinical diseases. Nevertheless, traditional platforms for EVs separation and detection platforms working alone often involve multiple pieces of equipment and complex, multi-step protocols. This extends processing time and the likelihood of bioanalyte loss and cross-contamination, thereby impeding further EVs research. To date, few studies have effectively combined EVs separation, detection, and analysis functions into a single platform. Integrated microfluidic platforms present a compelling solution by enabling seamless progression from sample to result. These platforms can efficiently combine various separation and detection techniques, simplifying complex workflows and facilitating both efficient EVs separation and high-sensitivity detection. This review concentrates on integrated microfluidic platforms for EVs separation and detection, specifically examining whether the separation and detection units are fully integrated. Recent studies underscore the potential of EVs as promising biomarkers for early-stage diagnosis of diseases, including cancer and neurodegenerative disorders. Recent advances in EVs separation and analysis enable overcoming key translational barriers, accelerating their routine adoption in clinical diagnostics.

## I. INTRODUCTION

### A. EVs: Biogenesis and clinical potential

**FIG. 1.**
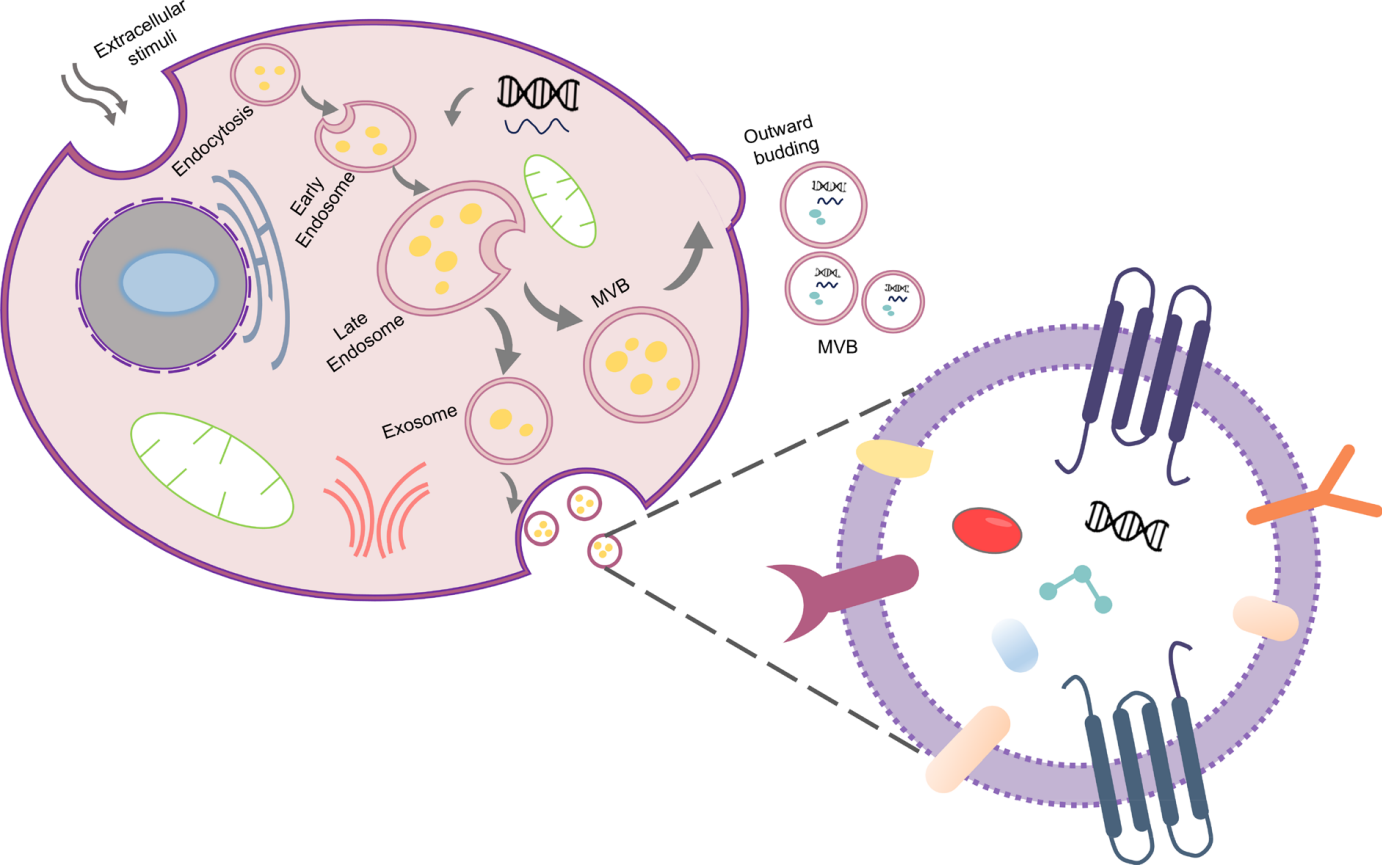
Biogenesis process of EVs and schematic diagram of the molecular composition of EVs.

Extracellular vesicle (EVs; with diameters ranging from 30 to 150 nm) represent a specialized subtype of EVs, which are membrane-enclosed entities synthesized by living cells via the processes of endocytosis, fusion, and exocytosis.^1^ EVs are categorized into apoptotic vesicles (>1 *μ*m), microvesicles (100 nm to 1 *μ*m), and EVs (30 to 150 nm).^2,3^ EVs are instrumental in the stabilization and transfer of cellular cargo, encompassing specific nucleic acids, functional proteins, and lipids, thereby enhancing intercellular communication.^4^ The biogenesis of EVs is a multifaceted process: initially, the plasma membrane undergoes invagination to generate early sorting endosomes (ESEs). These ESEs subsequently mature into late sorting endosomes (LSEs), which further invaginate to form multivesicular bodies (MVBs).^5,6^ Ultimately, MVBs are released into the extracellular milieu, transforming into EVs^7^ (Fig. 1). EVs are abundantly found across various biological fluids, including blood, urine, saliva, breast milk, lymphatic fluid, and cerebrospinal fluid.^8^ Recent investigations reveal that EVs are significantly more prevalent in the blood of cancer patients compared to healthy individuals. They are implicated in pivotal processes such as oncogenesis, disease progression, metastasis, and therapeutic monitoring.^9^ For instance, pancreatic cancer patients show elevated levels of glypican-1 EVs in blood, correlating with tumor burden.^10^ Moreover, EVs have emerged as biomarkers for evaluating therapeutic responses in cancer treatment and serve as indicators in liquid biopsies,^11^ underscoring their potential as highly effective instruments for minimally invasive diagnostics and early screening in preventive healthcare.

### B. Limitations of conventional EVs analysis

A range of conventional techniques for the analysis of EVs, including separation, imaging/morphological characterization, molecular profiling, and clinical application, have been progressively developed. However, these methods still face notable limitations that hinder their broad clinical translation. Achieving high-purity and efficient EVs separation from complex biological samples remains a major challenge in the separation stage. Although ultracentrifugation is widely regarded as the gold standard,^12^ it is time-consuming and labor-intensive, requires large sample volumes and expensive equipment, and may inadvertently activate platelets or immune cells,^13^ thereby compromising EVs purity and downstream analyses. For imaging and morphological characterization, high-resolution techniques such as transmission electron microscopy (TEM) and scanning electron microscopy (SEM) require elaborate sample preparation and are unsuitable for high-throughput applications.^14^ Alternative methods such as dynamic light scattering (DLS) and nanoparticle tracking analysis (NTA) are commonly used to measure particle size and concentration, but they lack sufficient resolution to distinguish heterogeneous EVs populations.^15^ In the molecular analysis stage, EVs surface markers (e.g., CD9, CD63, and CD81) are typically identified using Western blotting or enzyme-linked immunosorbent assay (ELISA).^16^ Although these methods provide specificity, their sensitivity is limited, and quantitative performance remains suboptimal. In summary, although conventional EVs separation and detection methods are valuable for basic research, their reliance on complex instrumentation and multi-step workflows limits their suitability for clinical diagnostics, which require high throughput, speed, and cost-effectiveness.

### C. Microfluidics as an integrated solution

Microfluidic technology, as an integrated and multifunctional platform, spans and optimizes the entire workflow of EVs analysis and is increasingly emerging as a powerful tool to overcome the limitations of conventional methods.^17–19^ These limitations underscore the need for integrated platforms that combine high purity, sensitivity, and scalability—criteria increasingly met by microfluidic technologies. In the separation stage, microfluidic devices can achieve high-throughput and highly specific EVs extraction through physical, chemical, or immunological mechanisms.^20,21^ For imaging and morphological characterization, microfluidic chips are typically fabricated from transparent materials and designed to be compatible with various microscopy systems, enabling *in situ* visualization and analysis of EVs.^22^ At the molecular analysis level, microfluidic platforms can integrate multiple sensing modules for the rapid and highly sensitive detection of functional biomolecules such as EV-associated proteins and RNAs.^23,24^ Finally, in clinical applications,^25^ microfluidic systems offer advantages such as operational simplicity, low sample volume requirements, and cost-effectiveness, demonstrating strong potential for clinical translation. While microfluidics offers high throughput and integration, challenges remain in standardization and manufacturing scalability. Nevertheless, its ability to combine separation (acoustics, immunoaffinity) with on-chip detection (electrochemical sensors) makes it uniquely suited for clinical translation.^26^ The ability of microfluidics to integrate separation, detection, and analysis on a single chip makes it a highly promising and practical platform for rapid EVs analysis, addressing the diverse needs of both fundamental research and clinical diagnostics. Some comprehensive reviews have outlined microfluidics-based technologies for EVs separation and detection.^27–29^ In this review, we place particular emphasis on the integrated microfluidic platforms designed for EVs analysis. We discuss distinct objects of EVs analysis, with a focus on comprehensive EVs analysis, detailed analyses of EVs' proteins and RNA are also provided, using the frameworks of the “combined microfluidic platform” and the “shared microfluidic platform” as foundational models.

## II. MICROFLUIDIC EVs SEPARATION AND DETECTION STRATEGIES

Microfluidics entails the precise manipulation of microscale fluid volumes within intricately designed micro- and nanostructured channels.^30^ This advanced technology facilitates the seamless convergence of sample preparation, analytical detection, and diagnostic operations onto a singular microchip. Boasting key advantages such as minimal sample consumption, accelerated separation and purification processes, enhanced sensitivity, and superior separation efficiency,^7,31^ microfluidics has rapidly established itself as a formidable tool in the realm of biomedical research.

### A. Microfluidic EVs separation strategies

#### 1. Microfluidic EVs separation strategies

**FIG. 2.**
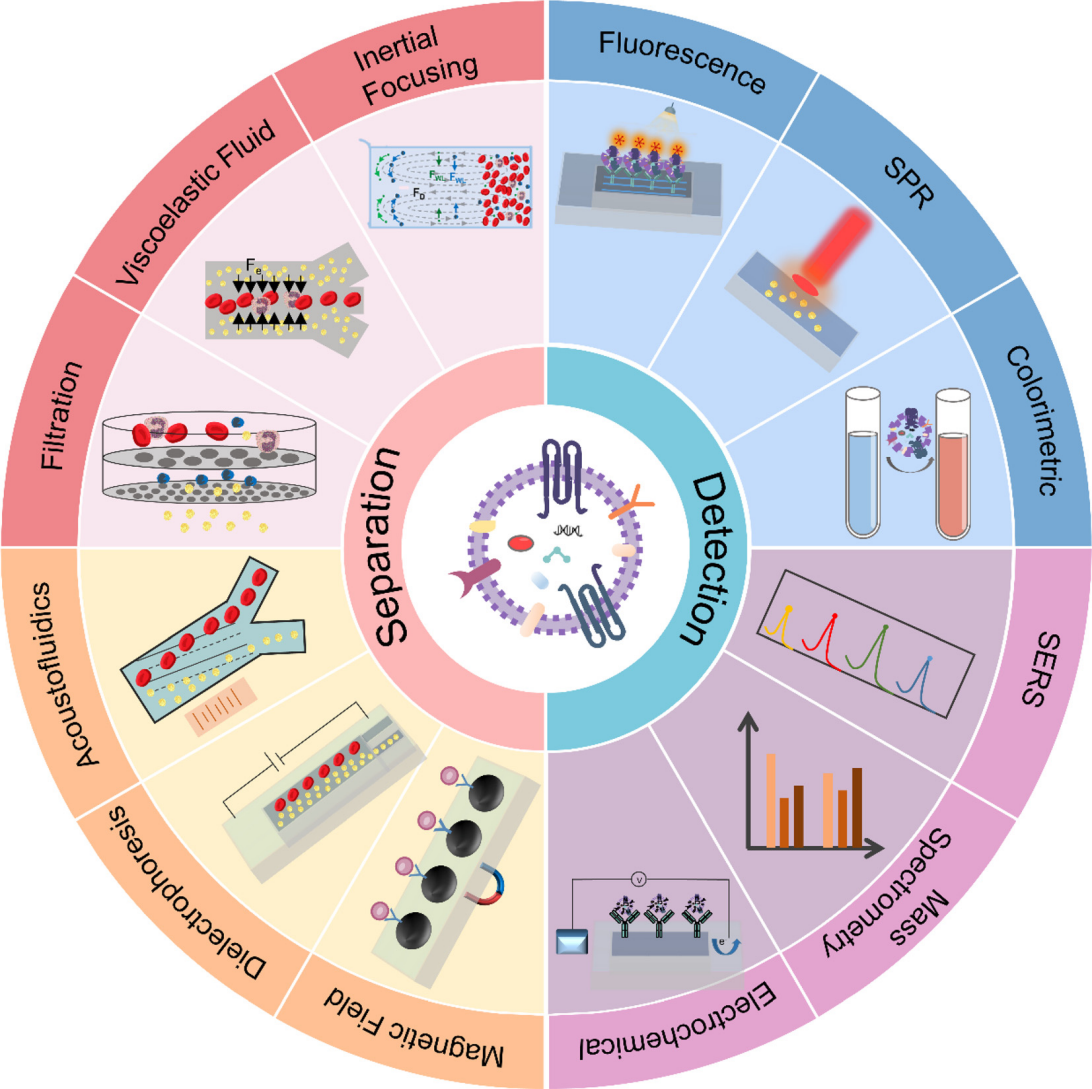
A schematic illustration of strategies for the separation and detection of EVs. The separation techniques, such as inertial focusing, viscoelastic fluid, filtration, acoustofluidics, dielectrophoresis (DEP), and magnetic field, are employed to separate EVs from various sample types. For detection, methods like fluorescence detection, surface plasmon resonance (SPR) detection, colorimetric detection, surface-enhanced Raman scattering (SERS), mass spectrometry-based detection, and electrochemical detection assays are utilized to further identify and analyze the separated EVs.

Microfluidics has made significant strides in EVs applications, praised for its efficiency in separation and detection.^32^ Yet, these claims often overlook challenges such as low throughput, inconsistent performance across biological samples, and scalability issues. Despite advantages like miniaturization and automation, microfluidics faces ongoing problems with reproducibility, complex integration, and the need for specialized expertise. Having said that, these microfluidic technologies effectively leverage both the physical characteristics of EVs, such as size and density, and their chemical features, including nucleic acids and surface proteins, by using microchip platforms to facilitate efficient EVs separation and detection.^33,34^ As a promising alternative to conventional methods, microfluidic-based approaches address current limitations through innovative integration strategies. The combination of techniques such as viscoelastic flow,^35^ acoustofluidics,^36^ dielectrophoresis,^37^ and magnetic field-based microfluidic systems is anticipated to greatly enhance the efficiency of EVs separation,^38^ enabling high-throughput, label-free separation of EVs directly from complex biological samples (Fig. 2). Table I summarizes various microfluidic-based strategies for exosome separation.

**TABLE I.** Summary of microfluidic EVs separation strategies.

Method	Throughput (*μ*l/min)	Recovery (%)	Scalability	Clinical feasibility	References
Inertial focusing	1.67	52.8 ± 4.53	High throughput and robustness; membrane-free system; ease of operation	Efficient submicrometer particle separation; integration with clinical testing	39
Viscoelastic fluids	3.34	87	Suitable for large-scale production; progress steadily	High recovery and purity; low operational cost and ease of use; high repeatability	40
Filtration	2.4	81	Low sample volume; high sensitivity and efficiency; quantification on a single chip	Direct EVs separation from blood; quantification of disease markers; fingertip-based testing	41
Acoustofluidics	10	99	Automation and high reproducibility; continuous flow configuration; adjustable separation parameters	Speed and high efficiency; preservation of EVs integrity; minimal user intervention	36
Dielectrophoresis (DEP)	30–50	NA	Rapid separation and recovery process; no sample dilution required; use of whole blood	Minimally invasive technology; on-chip biomarker detection; faster results for diagnostics	42
Magnetic separation	100	NA	Enhanced separation efficiency; high sensitivity and low contamination; reversibility and expandability	Efficient separation of T-EVs; minimized contamination; integration with mass spectrometry	43

Inertial focusing, a specialized form of hydrodynamic focusing, leverages the interplay between Dean forces and inertial lift forces exerted on particles of varying sizes within microfluidic channels under the influence of Newtonian fluids.^44,45^ The equilibrium of these forces generates distinct equilibrium positions along the curved channel, enabling size-dependent sorting of particles.^46,47^ Tay *et al.* developed a spiral microchannel system for the rapid purification of submicrometer particles directly from whole blood, achieving a recovery rate of 52.8 ± 4.53% for particles smaller than 1 *μ*m, with a reported resolution approaching 1 *μ*m.^39^ To further improve scalability, the authors later introduced an inertial microfluidic system based on previous work, enabling the direct separation of nanoscale EVs (exosomes, 50 to 200 nm) and medium-sized EVs (microvesicles, 200 nm to 1 *μ*m) from whole blood.^48^ That said, its sensitivity to nanoscale particles is limited due to inherently weak inertial forces at that scale. As a result, effective separation of small EVs remains challenging and often requires extended channel lengths to generate adequate Dean flow.^49^ This trade-off between efficiency and system complexity raises concerns about scalability, particularly for clinical applications requiring rapid, high-throughput EVs processing.

Viscoelastic fluids enable particle separation by exploiting the elastic lift forces generated by the medium's viscoelastic properties,^50–53^ typically composed of synthetic polymers such as polyvinylpyrrolidone (PVP), polyethylene oxide (PEO), and polyacrylamide (PAA).^54^ Kim *et al.* first demonstrated the use of viscoelastic flow in straight microchannels to focus 500 and 200 nm particles, marking a key step toward EVs separation using this mechanism, despite the inability to focus 100 nm particles.^55^ Building on this, Meng *et al.* developed a cascaded viscoelastic microfluidic device that enabled high-purity (>97%) and high-recovery (>87%) separation of small extracellular vesicles (sEVs, <200 nm) directly from whole blood.^40^ The device achieved a 100 nm cutoff size and a throughput of 3000 *μ*l/min, enabling rapid and nondestructive EVs extraction. While promising, viscoelastic microfluidics still faces challenges, including limited fluid stability, reduced reproducibility under high-throughput conditions, and poor scalability. Future efforts should aim to improve flow stability, control particle trajectories more precisely, and facilitate clinical translation through chip-level optimization.

Filtration leverages membranes with precisely defined pore sizes to selectively retain particles larger than the membrane pores, while permitting smaller particles, such as EVs, to pass through, thereby enabling size-based separation.^56–58^ This method can be classified into three primary types: membrane filtration, column filtration, and ultrafiltration (UF).^59–61^ Chen *et al.* developed a microfluidic platform for EVs separation from whole blood using dual membrane filtration, incorporating a 0.2 *μ*m polycarbonate membrane to enhance performance.^41^ The system achieved over 99% sEV separation efficiency and an 81.1% recovery rate from just 2 *μ*l of blood. Despite its high separation efficiency, the device faced limitations such as membrane clogging and potential EVs damage. To overcome these issues, Li *et al.* introduced a centrifugal microfluidic system combining dual nanofilters with pneumatic pulse filtration. This pulsatile flow reduced clogging and particle deposition, enabling EVs separation within 30 min and yielding 91% diagnostic accuracy for early breast cancer.^62^ Filtration-based methods offer simplicity and high purity but typically require external forces such as centrifugation, pressure, or vacuum to drive fluids through membrane pores. As samples concentrate, membrane fouling becomes more likely, reducing efficiency. Additionally, dependence on external actuation hinders integration into clinical workflows.

Acoustofluidics integrates external acoustic fields with microfluidic frameworks to actively sort particles by size,^63–65^ offering significant advantages in both precision and biocompatibility.^66–68^ Wu *et al.* developed an acoustofluidic device employing tilted surface acoustic waves (TSAWs) for continuous-flow exosome separation, achieving a recovery rate of approximately 90%.^69^ They later introduced a rapid, label-free two-module system, where the first module removed blood cells while maintaining an EVs recovery rate above 99%, and the second module purified EVs with a reported purity of 98.4%.^36^ An additional acoustic module was subsequently integrated to further improve performance. These results underscore the potential of acoustic fields for efficient, high-purity EVs separation. However, challenges remain, including frequent air bubble formation within the acoustic field and high system costs, which may hinder clinical translation. Improving device stability, reducing system complexity, and validating clinical applicability in large-scale studies are essential next steps.

Dielectrophoresis (DEP) refers to the migration of particles in a non-uniform electric field, where differential forces induce particle or cellular polarization, generating a net dipole moment on the particle's surface.^70^ This polarization allows the particles to move either along or counter to the electric field, contingent upon factors such as excitation frequency, particle size, and electrode configuration.^37^ Lewis *et al.* employed an alternating current electrokinetic (ACE) microarray chip to separate EVs via DEP. In this system, EVs were concentrated in high-field regions around circular microelectrodes, while larger cells were excluded into low-field zones. The device enabled direct EVs enrichment from 30 to 50 *μ*l of whole blood without prior sample processing.^42^ It was later optimized for *in situ* immunofluorescent labeling and surface protein analysis on-chip.^10^ Although the method showed high enrichment efficiency, the recovery rate was not reported, raising concerns about overall yield and sample loss. DEP is appealing for its ability to selectively manipulate EVs using tunable electrical parameters, such as frequency and voltage. Nonetheless, it still faces challenges, including low throughput and the requirement for low-conductivity buffers, which may limit compatibility with biological fluids. Improving scalability and operational stability will be essential for translating DEP-based EVs systems into clinical use.

Magnetic separation involves the capture of EVs through an immunoaffinity reaction, wherein magnetic beads (MB) bind to antibodies. This process utilizes magnetic forces generated by a magnetic field to manipulate the particles effectively.^71,72^ Niu *et al.* developed the develop a fluid multivalent magnetic interface (FluidmagFace) in a microfluidic chip, a system that employs affinity magnetic beads (MB) for efficient EVs separation and proteomic analysis. Magnetic separation allows precise manipulation of MB-bound targets, improving separation efficiency by 13.9% compared to non-bivalent binding methods.^43^ This technique effectively and selectively captures EVs from plasma samples with high sensitivity. While magnetic separation offers excellent affinity and selectivity, its effectiveness can be affected by external interference, limiting reliability in complex biological samples. Although the technique demonstrates promising sensitivity and specificity, overcoming the impact of environmental factors remains a critical challenge for its broader clinical implementation.

Building on previous research, the proposed platform demonstrates considerable promise for clinical applications, particularly in areas such as noninvasive liquid biopsy and personalized medicine. By enabling the detection and analysis of tumor-derived EVs (T-EVs) in blood, the platform facilitates early cancer diagnosis and continuous monitoring of treatment responses.^43^ Moreover, it offers the potential to identify biomarkers associated with drug resistance, thereby aiding in the customization of cancer treatments. Beyond oncology, the system's ability to separate and analyze circulating microparticles (MPs) provides a valuable diagnostic tool for assessing vascular health, offering insights into conditions such as atherosclerosis and diabetes-related cardiovascular risks.^73,74^

From a clinical adoption perspective, while acoustofluidics requires a higher initial investment due to the specialized equipment involved, it offers substantial long-term cost savings.^75^ Its automated, label-free operation reduces the need for consumables and manual labor, making it more cost-efficient over time compared to techniques like ultrafiltration, which are burdened by ongoing costs for membrane replacements and maintenance. As the system scales for high-throughput applications, the per-test cost of acoustofluidics becomes more favorable, further enhancing its potential for widespread clinical use. This cost comparison underscores the economic advantages of acoustofluidics, positioning it as a promising solution for clinical adoption in both oncology and vascular health diagnostics.^76^ Current comparisons favor older benchmarks; emerging alternatives require direct validation. Multicenter reproducibility studies remain imperative before cost advantages can be deemed generalizable.^77,78^

#### 2. Purity metrics in EVs separation strategies

A significant challenge in microfluidic-based EVs separation is the lack of standardized metrics for assessing EVs purity, which hinders the ability to directly compare the performance of different platforms. Purity is typically quantified by evaluating the presence of contaminants, such as lipoproteins and soluble proteins, which may co-isolate with EVs during the separation process.^79^ However, the methods used to assess purity vary widely across studies, affecting the reported purity values and their interpretability. For instance, acoustofluidic separation techniques have reported a purity of 98.4%, typically by excluding lipoproteins from the analysis.^36^ This step is crucial for ensuring the specificity of the isolated EVs population. However, some studies fail to fully detail the specific methods employed to exclude lipoproteins and address the potential impact of other co-isolated components.^80,81^ A more thorough explanation of the purity quantification process, including the methods used to remove lipoproteins and non-EVs contaminants, is essential for ensuring accurate purity assessments.

In contrast, the viscoelastic flow-based method reports a purity of greater than 97% yet does not account for the potential co-isolation of soluble proteins.^40^ While soluble proteins are not directly associated with EVs, their presence can influence the overall purity of the isolated EVs.^82^ This issue complicates the interpretation of purity measurements and highlights the need to consider all potential contaminants when evaluating EVs separation techniques.

Microfluidic technologies have demonstrated significant potential in efficiently separating EVs from complex biological fluids.^83^ Nonetheless, a major challenge remains in achieving high purity of the separated EVs, particularly due to the co-separation of contaminants such as lipoproteins and soluble proteins. These contaminants can compromise the integrity and specificity of the EVs population, thus undermining the reliability of subsequent analyses. Therefore, addressing these sources of contamination is essential for enhancing the performance of microfluidic EVs separation techniques. Several microfluidic approaches, including acoustofluidics and viscoelastic flow-based methods, have been developed to improve EVs separation purity.^26,84^ Acoustofluidics can effectively minimize lipoprotein contamination by adjusting the acoustic field parameters to preferentially capture EVs while reducing the co-separation of larger lipid particles.^85^ However, despite achieving high purity by excluding lipoproteins, acoustofluidic systems may still face difficulties in separating EVs from soluble proteins, which share similar size and density characteristics.^86^ To further mitigate this issue, incorporating additional steps such as affinity-based capture or size-exclusion filtration may be necessary to reduce soluble protein contamination.

### B. Microfluidic EVs detection strategies

We reviewed microfluidic strategies for EVs separation, emphasizing that accurate downstream detection relies on EVs being separated with high purity.^87^ Various EVs detection techniques have been used in disease prognosis and therapeutic monitoring. Microfluidic methods such as fluorescence assays, SPR, SERS, mass spectrometry, and electrochemical sensing offer benefits in miniaturization and integration (Fig. 2). Nevertheless, they often require expensive equipment and high reagent volumes and may lack sensitivity for detecting low-abundance biomarkers.^88–92^ Nonetheless, rapid advances in microfluidic technology are driving its use in EV-based diagnostics. These platforms show strong potential for high-throughput, sensitive detection from small sample volumes. Future work should improve robustness, sensitivity, and clinical adaptability while addressing standardization and reproducibility to enable broader clinical use.

Fluorescence detection, known for its high precision and sensitivity, offers a rapid and efficient method for EVs detection, particularly when combined with microfluidic platforms.^87^ EVs are typically captured on microfluidic chips and labeled with fluorescent dyes or quantum dots.^93–95^ The resulting signals are analyzed via fluorescence microscopy or flow cytometry, with their presence and intensity serving as diagnostic indicators.^96^ Chinnappan *et al.* developed an aptamer-based magnetic biosensor using magnetic nanobeads functionalized with anti-CD63 aptamers. By integrating immunomagnetic separation (IMS), the system enhanced fluorescence detection of EVs, with CD63 serving as the target marker. Carbon-coated magnetic beads adsorbed FAM (6-Carboxyfluorescein)-labeled ssDNA aptamers, resulting in a strong fluorescence signal and a detection limit of 1457 particles/ml.^97^ Fluorescence-based methods enable rapid and sensitive EVs detection and hold promise for *in situ* analysis of clinical samples. However, their clinical translation is limited by nonspecific binding, high cost, and complex procedures. Additionally, the small size and heterogeneity of EVs challenge labeling consistency and quantification. Quantum dots (QDs) offer excellent photostability but pose toxicity risks due to heavy metals like cadmium and lead, limiting their clinical use.^98,99^ Biodegradable alternatives, such as fluorescent nanoparticles from organic dyes or biocompatible polymers, provide similar optical properties with enhanced safety profiles. These materials can also be engineered to degrade in specific biological environments, improving biocompatibility.^100^ Exploring these options could lead to safer, more reliable biosensing platforms for applications like EVs analysis and biomarker detection.

Surface plasmon resonance (SPR) is an advanced optical biosensing technology that exploits the phenomenon of total internal reflection of light at the interface between a prism and a metal film.^101^ This interaction generates a diminishing wave within a photophobic medium, alongside a plasmonic wave present in the medium.^102^ Luo *et al.* developed a portable SPR-based EVs sensing platform that integrates microfluidics with a high-performance trilayer structure: a gold mirror, SiO_2_ spacer, and nanoporous gold sensing layer. The SiO_2_ layer served as an optical cavity, and the sensor surface was functionalized with CD63 or EpCAM aptamers for EVs detection. The average bulk sensitivity values for the Si/Au hole, Au/Au hole, and CIMH sensors were 316, 391, and 431 nm/RIU, with corresponding FOM values of 1.93, 12.1, and 29.2, respectively.^103^ This platform achieved a limit of detection of 6 × 10^5^ particles/ml, demonstrating its potential for ultrasensitive EVs analysis. While SPR offers advantages in label-free, real-time detection for clinical diagnostics, its widespread use is hindered by the complexity, cost, and size of current systems. To unlock its full clinical potential, miniaturization, system integration, and simplified operation are critical.

Colorimetric detection is a method for identifying exosomes through a color change in solution, leveraging the simplicity of operation and signal visualization. This approach is integrated with a microfluidic platform to enhance its efficiency and practicality.^104,105^ Chen *et al.* developed a ZnO nanowire-coated scaffold chip for colorimetric detection of EVs using TMB, achieving a detection limit of 2.2 × 10^7^ particles/ml.^105^ While simple, colorimetric detection often requires complementary methods to enhance sensitivity. Vaidyanathan *et al.* introduced a tunable AC hydrodynamic technique that utilizes nanoshear forces to improve exosome capture and detection, with a fivefold increase in efficiency, detecting over 2.76 × 10^6^ particles/ml.^106^ That said, both methods face challenges, including reaction conditions, matrix interference, and nonspecific binding. Improvements in scalability, robustness, and reproducibility are crucial for clinical application, with future efforts focusing on high-throughput, clinical-grade platforms.

Surface-enhanced Raman scattering (SERS) is a sophisticated vibrational spectroscopy technique that amplifies Raman signals through plasmon excitation on irregular metal surfaces, predominantly gold (Au) or silver (Ag).^107^ SERS offers highly sensitive detection of low-concentration analytes,^108–110^ and can be seamlessly integrated into microfluidic systems for high-throughput and precise analysis.^111^ To achieve highly sensitive detection of EVs, Lee *et al.* developed an SERS substrate with gold nanopillars that generate hotspots, enabling precise miRNA detection of EVs at detection limits over 100 times lower than other methods; the detection sensitivity range is 1 aM to 100 nM.^112^ This was accomplished using a locked nucleic acid probe. While SERS offers exceptional sensitivity and label-free detection, challenges persist, including unstable signal enhancement, background noise, and high equipment costs. Future efforts should focus on improving signal stability, reducing noise, optimizing cost and scalability, and developing more robust substrates with better system integration for clinical applications.

As an integral component of proteomics, mass spectrometry offers several advantages in bioassays, including high throughput, exceptional specificity, remarkable sensitivity, and cost-effectiveness.^113,114^ Consequently, it has been extensively employed in the analysis of EVs' proteins and amino acids. Shan *et al.* developed a matrix comprising gold nanoparticles (AuNPs) and cellulose nanocrystals (CNCs) for the direct analysis of full proteins in serum-derived EVs, effectively addressing ion suppression caused by protein aggregation.^115^ This method reduced the detection limit to 0.01 mg/ml, enhancing sensitivity, automation, and cost-efficiency, and enabling the detection of a wide range of proteins with excellent reproducibility. The specificity of this detection method reaches 83.2%. However, the high cost of AuNPs, the complexity of sample preparation, and the challenges associated with data analysis hinder their broader clinical adoption. Future advancements should focus on streamlining sample preparation, lowering costs, and simplifying data analysis to facilitate more widespread clinical application.

Electrochemical detection relies on electrochemical reactions that convert sample concentration and structure into measurable electrochemical potentials.^116,117^ This method is straightforward, efficient, and highly selective, allowing for the quantification of EVs through alterations in electrochemical signals. Jiang *et al.* developed a poly(dopamine) (PDA)-assisted aptamer-based DNA microelectrode sensor for electrochemical EVs detection. The PDA deposition enhances the surface complexity, significantly improving both sensitivity (81.9%) and precision. Utilizing electrochemical impedance spectroscopy (EIS), the sensor achieves a detection limit of 1.39 × 10^2^ particles/ml (approximately 14 EVs) within 180 min, enabling single-particle detection.^118^ While this method shows great promise for early cancer diagnosis, challenges remain, particularly with electrode lifespan and the potential economic burden of frequent replacements. Future research should focus on extending the electrode lifespan, enhancing system integration, and addressing reproducibility issues to facilitate broader clinical adoption.

Meanwhile, emerging techniques, such as digital ELISA and nanopore-based EVs counting, are transforming the field of EVs detection by providing exceptional sensitivity and resolution. Digital ELISA, with its attomolar sensitivity, enables the detection of low-abundance biomarkers within complex biological samples, making it particularly advantageous for early disease diagnosis and continuous monitoring.^95,119–122^ In contrast, nanopore-based EVs counting offers single-particle resolution, allowing for precise enumeration of individual EVs and the detailed characterization of their heterogeneity.^123^ The integration of these advanced technologies with microfluidic platforms has the potential to overcome current limitations in both sensitivity and scalability. By combining the high-throughput capabilities of microfluidics with the superior sensitivity of digital ELISA and the single-particle resolution of nanopore-based counting, it becomes possible to significantly enhance the efficiency and accuracy of EVs analysis.^124,125^ This integration could thus facilitate more reliable diagnostic tools and enable more effective personalized medicine applications.

## III. INTEGRATED MICROFLUIDIC PLATFORM FOR EVs ANALYSIS

**FIG. 3.**
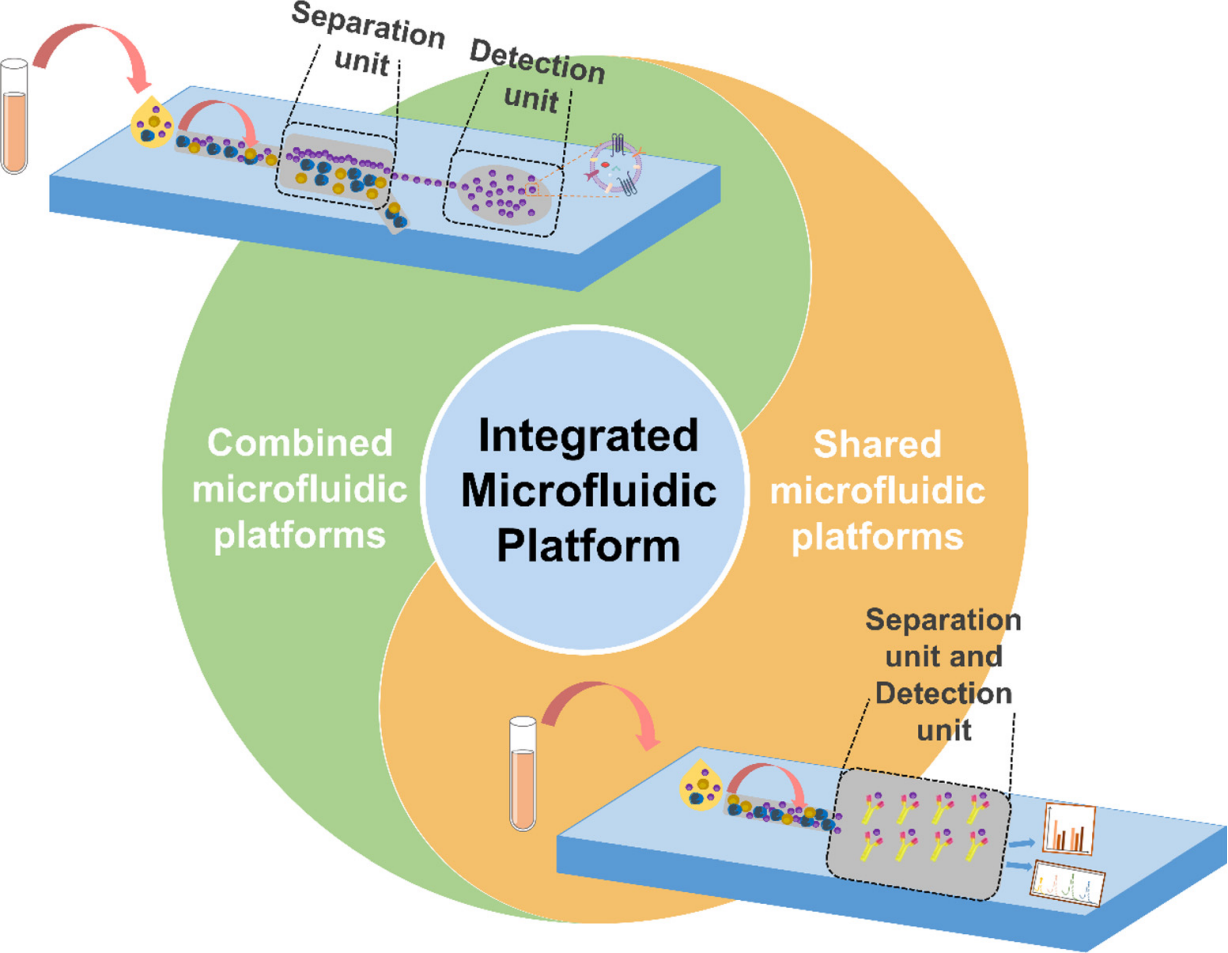
A schematic representation of combined and shared microfluidic platforms built on integrated microfluidic systems. In the combined microfluidic platform, the separation and detection regions are mechanically linked via microchannels or tubing, enabling continuous operation. EVs are first separated in the separation region and then directed downstream to the detection region. In contrast, the shared microfluidic platform lacks a distinct detection area; here, EVs are both separated and captured within the separation region, where detectors are directly applied for *in situ* analysis of the EVs.

Building upon the previous systematic review of strategies for the separation and detection of EVs, significant advancements have been made in the methodologies employed for EVs separation and analysis. The microfluidic separation platform has evolved to enhance EVs extraction, achieving both high yield and exceptional purity, while detection modalities continue to diversify. Nevertheless, many studies tend to apply separation and detection techniques independently.^126,127^ This fragmented approach complicates experimental protocols and significantly increases both time and costs, ultimately hindering research efficiency and limiting practical applicability.^128^ Recently, many researchers have shifted their focus toward integrating separation and detection systems, leading to significant advancements in both sample preparation and direct analytical evaluation. As summarized in this review, current integration strategies can be primarily categorized into two paradigms: the “combined microfluidic platform” and the “shared microfluidic platform” (Fig. 3). The combined microfluidic platform uses a serial configuration of separation, enrichment, and detection units interconnected via microchannels or pipelines, facilitating the continuous operation of both enrichment and detection processes. In contrast, the shared microfluidic platform eliminates the need for an additional detection system, enabling enrichment and detection functionalities to operate on a single platform, supporting *in situ* and real-time EVs analysis. Furthermore, depending on the specific requirements for subsequent EVs analysis, detection methods across various integrated platforms can be further classified into comprehensive content analysis and bioactive substance profiling, which include the assessment of proteins and nucleic acids. Table II provides a comparison of combined microfluidic platforms and shared microfluidic platforms for EVs. Evaluating long-term operational costs of the combined and shared microfluidic platforms involves factors like consumables, maintenance, and scalability. The combined platform, with its modular design, incurs higher consumable and maintenance costs due to additional reagents and frequent calibration. This can limit its feasibility in resource-limited settings. In contrast, the shared platform has lower initial consumable costs but requires careful optimization to prevent process interference. Maintaining high performance over time may lead to higher maintenance costs.

**TABLE II.** Comparison of combined microfluidic platforms and shared microfluidic platforms for EVs.

Type	Merits	Demerits	Clinical suitability
Combined microfluidic platforms	High flexibility; multi-step optimization; precise analysis	Increased complexity; higher cost; slower throughput	Suitable for detailed; high-precision analyses; less ideal for rapid, point-of-care diagnostics
Shared microfluidic platforms	Streamlined; cost-effective; real-time analysis	Lower flexibility; limited advanced detection options	Ideal for rapid; point-of-care diagnostics and real-time monitoring

### A. Combined microfluidic platforms for EVs analysis

Following the separation process of tumor-derived EVs, the integrated system typically evaluates these EVs based on three key biophysical and biochemical characteristics: quantification (EVs count) and the analysis of bioactive molecules. A prevalent method for counting EVs involves correlating their accumulation on the chip with the quantification of fluorescence intensity.^95,129^ Furthermore, individual EVs can be encapsulated and separated for subsequent counting analyses. The application of microfluidics technology in the detection of individual EVs holds significant promise, particularly when integrated with advanced analytical techniques such as digital droplet technology [including digital PCR (dPCR) and digital ELISA] and nanoarray technology.^95,119–122^ Over the past few years, numerous scholars have dedicated themselves to the development of combined microfluidic platforms.^130–132^ Table III provides an overview of various combined microfluidic platforms.

**TABLE III.** Comparison of combined microfluidic platforms for EVs.

Platform	Separation technique	Detection technique	Sample	Limit of detection (LOD)	Work time	References
ExoPCD chip	Y-shaped microcolumn array	Electrochemical detection	Serum	4.39 × 10^3^ particles/ml	3.5 h	133
Apta-magnetic biosensor	Immunomagnetic bead separation	Fluorescence detection	Cell culture supernatant	1457 exosomes/ml	NA	97
ExoELISA	Droplet-encapsulated single exosomes	Fluorescence counting	Cell culture supernatant	10 exosomes/*μ*l	NA	95
EXID system	Serpentine microchannel	Microcolumn immunocapture assay	Blood	10.76 exosomes/*μ*l	2 h	134
EVLET	Vibrating membrane filtration (VMF)	Thermophoretic amplification	Plasma	4.1 × 10^5^ EVs	100 min	135
ExoSD chip	Immunomagnetic nanoparticles (IMNPs) separation	Fluorescence detection	Serum	NA	NA	71
ExoDEP chip	Microsphere-mediated dielectrophoretic separation	Fluorescence detection	Cell line supernatant	193 exosomes/ml	NA	130
PS-ED chip	Inertial focusing	Antibody capture	Whole blood	95 particles/*μ*l	NA	132
FEMC	Filtration	Electrochemical detection	Serum	1 × 10^4^ particles/ml	1 h	136
HiMEc	Inertial focusing	Electrochemical detection	Plasma	1 × 10^4^ to 1 × 10^8^ EVs/ml	NA	137
Nanomixing-microchip and SERS barcoding system	Nanomixing-microchip	SERS detection	Serum	NA	NA	138
Microfluidic-SERS method	Filtration	SERS detection	Plasma	2 EVs/*μ*l	5 h	139
Nanochannel array membrane and immunocapture chip	Filtration	Immunocapture detection	Serum	NA	1.5 h	140
Indium-tin-oxide (ITO) sensor	Multi-orifice flow-fractionation (MOFF) channel	Electrochemical detection	Plasma	15 EVs/*μ*l	NA	141
Double-filtration unit and photonic crystal (PC)	Filtration	Fluorescence detection	Serum	8.9 × 10^3^ EVs/ml	NA	142
PCR-free integrated microfluidics platform	Acoustofluidics	Concentration/sensing detection	Plasma	1 pM	30 min	24
Sample treatment and miRNA quantification module chip	Micromixer separation	dPCR-based miRNA quantification	Blood	11 copies/ml	4.3 h	143

#### 1. Combined platforms of overall EVs levels analysis

The comprehensive analysis of overall EVs levels is a crucial step in assessing the distribution and functionality of EVs *in vivo*. This analysis encompasses not only the absolute quantification of EVs but also their relative abundance and trends of variation across different biological samples.^144–146^ Commonly employed techniques include NTA, DLS, and FACS, all of which effectively provide insights into the size distribution and concentration of EVs. Microfluidic technologies have recently gained attention as complementary tools for EVs quantification, offering advantages such as low sample volume requirements, integration of multiple functions, and potential for automation.^147^ However, their sensitivity often varies with device design and surface chemistry, leading to inconsistent results. Furthermore, complex fabrication and operational requirements hinder standardization and reproducibility.

**FIG. 4.**
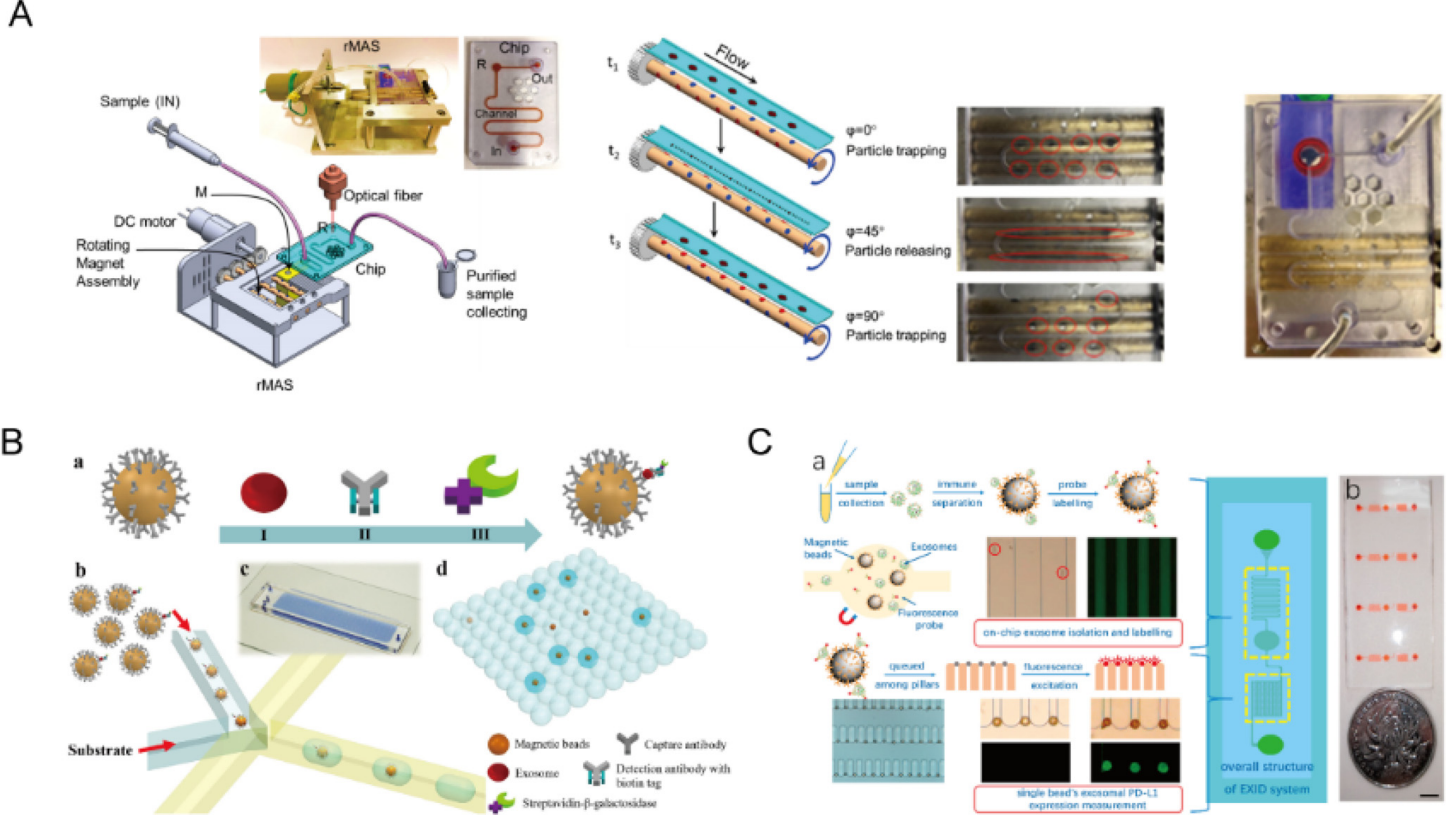
The combined platform was used for overall EVs content analysis. (a) An “apta magnetic biosensor” platform that utilizes anti-CD63 aptamers immobilized on the surfaces of magnetic nanobeads. Reprinted with permission from Chinnappan *et al.*, Biosens. Bioelectron. **220**, 114856 (2023). Copyright 2023 Elsevier.^97^ (b) The droplet digital ExoELISA microfluidic platform for single EVs counting and immunoassays. Reprinted with permission from Liu *et al.*, Nano Lett. **18**(7), 4226–4232 (2018). Copyright 2018 Authors, licensed under a Creative Commons Attribution License.^95^ (c) Immunomagnetic beads labeled with anti-PD-L1 fluorescent probes capture EVs, which are then separated within a serpentine channel before entering the microcolumn analysis area for single-bead analysis. Reprinted with permission from Lu *et al.*, Biosens. Bioelectron. **204**, 113879 (2022). Copyright 2022 Elsevier.^134^

The ExoPCD chip developed by Xu *et al.* offers a promising strategy for integrated EVs separation and electrochemical detection, achieving a low sample requirement (30 *μ*l) and a detection limit of 4.39 × 10^3^ particles/ml for CD63-positive EVs. Its two-stage design, which combines Y-shaped microcolumns for enrichment with a downstream electrochemical sensing region, enables *in situ* analysis and improves workflow efficiency.^133^ Tim4-PS-based magnetic enrichment further enhances specificity by reducing nonspecific capture. Yet, the platform's reliance on antibody-functionalized magnetic beads and a custom chip structure may pose challenges in terms of fabrication, cost, and reproducibility. Its focus on single-marker detection also limits adaptability compared to multiplexed or label-free platforms. Future improvements should aim to reduce processing time, expand marker coverage, and simplify device architecture. Incorporating machine learning-based signal analysis could further enhance robustness and clinical applicability. Chinnappan *et al.* introduced an “apta magnetic biosensor” platform featuring anti-CD63 aptamers immobilized on the surface of magnetic nanobeads [Fig. 4(a)]. The described method combines flow-through magnetic separation with immunomagnetic separation (IMS) for EVs separation, using a rotating magnet assembly system (rMAS) and aptamer-based fluorescence detection.^97^ While the system achieves a detection limit of 1457 EVs/ml and its sensitivity is promising, there are concerns about scalability and reproducibility. Additionally, while the rMAS and microfluidic channels improve automation, the complexity and cost of the system hinder its clinical adoption. Future developments should focus on improving scalability, robustness, and specificity, while simplifying the system for broader clinical use.^124,125^ Liu *et al.* developed a droplet-based microfluidic platform, termed droplet digital ExoELISA, for ultrasensitive immunoassays and single-EV counting [Fig. 4(b)]. By encapsulating CD63 antibody–conjugated magnetic beads within droplets and labeling breast cancer-derived EVs with GPC-1, the system achieves a detection limit as low as 1 × 10^4^ EVs/ml (∼10^−17^ M), demonstrating strong potential for early cancer diagnostics.^95^ Despite its high sensitivity, the platform faces notable challenges. The droplet generation process and magnetic bead manipulation introduce operational complexity and may affect reproducibility across laboratories. Additionally, reliance on antibody specificity can lead to cross-reactivity or inconsistent binding in heterogeneous samples. Compared to label-free or continuous-flow systems, droplet-based methods may also struggle with scalability due to precise flow control requirements. Future efforts should aim to streamline droplet handling, validate performance across diverse clinical samples, and incorporate multiplexing to broaden diagnostic applications. Lu *et al.* developed the EXID system, an integrated microfluidic platform combining serpentine channels and a microcolumn array for efficient EVs separation and detection [Fig. 4(c)]. Using anti-PD-L1-labeled immunomagnetic beads and quantum dot (QD)-based single-bead fluorescence analysis, the system enables multiplexed EVs profiling with a detection limit of 10.76 EVs/*μ*l and completes analysis within 2 h.^134^ This approach shows strong potential for tumor subtype classification and guiding immunotherapy. Nonetheless, the device's structural complexity may hinder large-scale manufacturing and workflow integration. Additionally, QD-based detection, while enhancing signal resolution, requires careful calibration and poses potential concerns regarding photostability and biocompatibility. The system's reliance on antibody specificity may also limit its reproducibility across heterogeneous EVs populations. To sum up, without addressing these technical and translational challenges, the promising analytical capabilities of current microfluidic platforms risk remaining confined to research settings.

#### 2. Combined platforms of EVs bioactive substances analysis

**FIG. 5.**
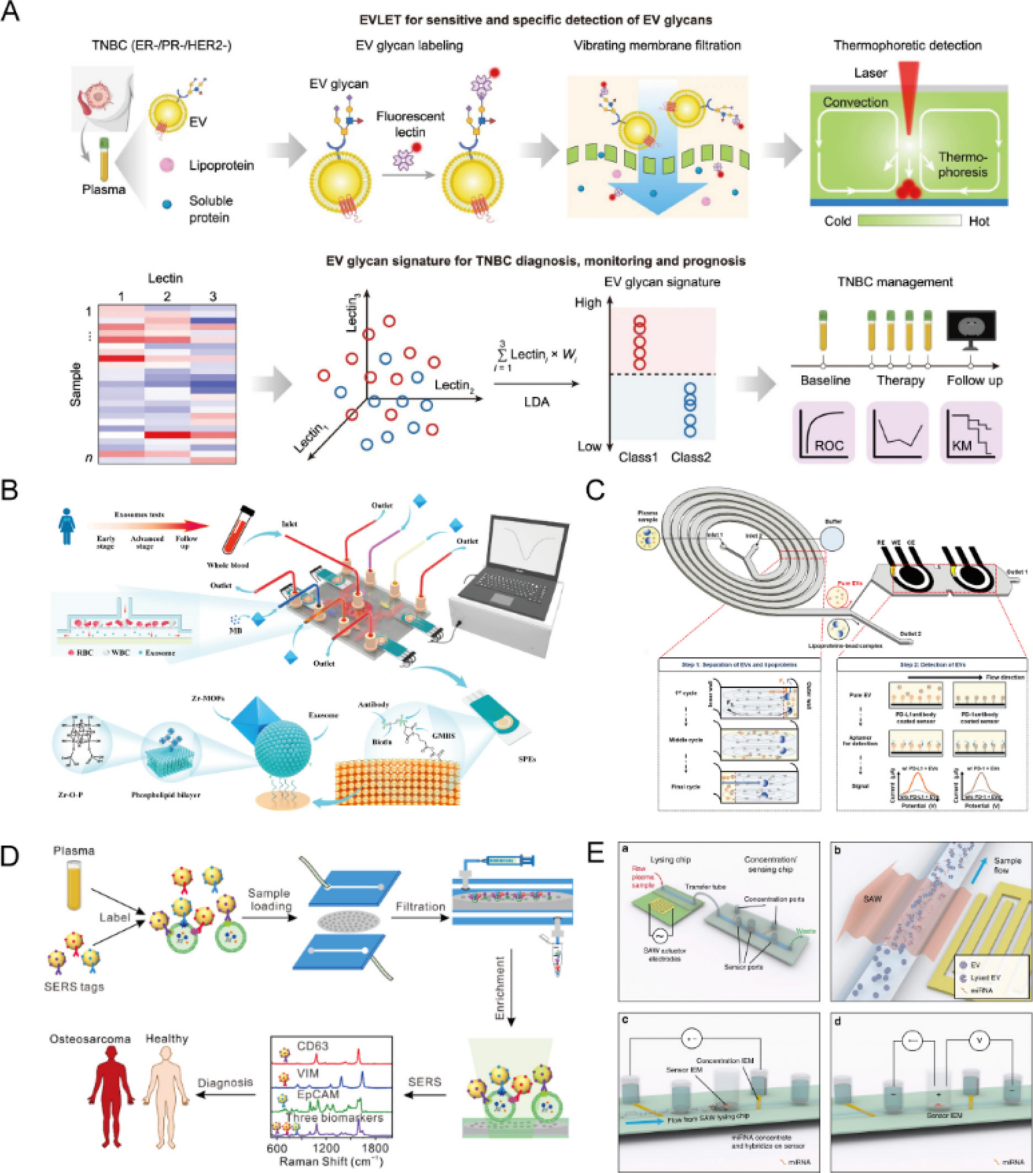
The combined platform was used for EVs bioactive substances analysis. (a) The lectin-based thermophoretic method (EVLET) streamlines the processes of vibrational membrane filtration (VMF) and thermophoretic amplification on a microfluidic platform for the analysis and detection of EVs glycans in cancer diagnostics. Reprinted with permission from Li *et al.*, Nat. Commun. **15**(1), 2292 (2024). Copyright 2024 Nature.^135^ (b) The dual-filter electrochemical microfluidic chip (FEMC) platform facilitates on-chip separation of EVs and continuous *in situ* electrochemical analysis of surface proteins. Reprinted with permission from Wang *et al.*, Biosens. Bioelectron. **239**, 115590 (2023). Copyright 2023 Elsevier.^136^ (c) An integrated platform for EVs separation and detection combining helical microfluidic channels with electrochemical devices (HiMEc). Reprinted with permission from Kwon *et al.*, Biosens. Bioelectron. **267**, 116792 (2025). Copyright 2025 Elsevier.^137^ (d) A microfluidic platform with integrated size filtration for SERS analysis of plasma from osteosarcoma patients. Reprinted with permission from Han *et al.*, Biosens. Bioelectron. **217**, 114709 (2022). Copyright 2022 Elsevier.^139^ (e) A microfluidic platform for EVs detection using integrated SAW lysis without PCR and chemical reagents. Reprinted with permission from Ramshani *et al.*, Commun. Biol. **2**(1), 1–9 (2019). Copyright 2019 Nature.^24^

The analysis of bioactive substances within EVs is a critical step in elucidating their roles in cellular communication, disease progression, and therapeutic interventions.^148,149^ The biomolecules transported by EVs, including proteins, nucleic acids (such as mRNA and miRNA), and glycans, participate in a myriad of physiological and pathological processes.^150,151^ Consequently, both quantitative and qualitative analyses of these molecules have become fundamental to understanding the functional dynamics of EVs. Currently, some research groups have utilized thermophoresis to target EVs glycans as detection objects, thereby breaking away from conventional methods of EVs detection and analysis [Fig. 5(a)].^135^

##### a. EVs' protein analysis.

The in-depth investigation of EVs proteomics lays a foundational framework for the discovery of potential EVs assays, which can be employed for early disease diagnosis and therapeutic monitoring.^152^ By comparing the EVs' proteome of healthy individuals with those suffering from various diseases, researchers have identified characteristic proteins associated with disease progression, thereby informing the development of personalized treatment strategies.^153^

Wang *et al.* developed a dual-filter electrochemical microfluidic chip (FEMC) that integrates EVs separation and *in situ* detection into a streamlined workflow [Fig. 5(b)]. By combining dual filtration with multiplexed screen-printed electrodes (SPEs) functionalized with MB@UiO-66 and specific antibodies, the platform enables rapid detection of multiple tumor markers (PMSA, EGFR, CD81, and CEA) from blood samples.^136^ It achieves a detection limit of 1 × 10^4^ particles/ml within 1 h, offering a 100-fold sensitivity improvement over traditional ELISA. Despite its advantages, the platform faces practical challenges. The use of multiple SPEs and pre-functionalized materials increases fabrication complexity and cost, potentially limiting scalability. Moreover, while MB@UiO-66 enhances signal output, it may introduce background noise if not properly controlled across diverse biological fluids. Although effective for protein markers, the platform's applicability to other biomolecules like RNA or lipids remains unverified, which could restrict its diagnostic versatility. Kwon *et al.* developed the HiMEc platform, integrating helical microfluidic channels with electrochemical sensors for efficient EVs separation and detection [Fig. 5(c)]. By combining size-based sorting with antibody-mediated lipoprotein capture, the system removes over 83% of lipoproteins and achieves an EVs recovery rate above 87%. Quantification of PD-L1 and PD-1 on EVs showed signal intensities 2.3× and 1.2× higher than those obtained via ultracentrifugation and SEC (size exclusion chromatography), respectively, with a detection range from 1 × 10^4^ to 1 × 10^8^ EVs/ml.^137^ Despite its strengths, HiMEc's structural complexity may limit scalability and standardization. Furthermore, its current design focuses on protein biomarkers, with limited validation for nucleic acid detection. While it offers enhanced purity and sensitivity compared to conventional methods, further optimization is needed to improve usability, broaden analytical targets, and ensure reproducibility across clinical samples. To enhance detection sensitivity and efficiency, integrated microfluidic platforms utilizing SERS offer notable advantages,^108–110^ enabling high-throughput and sensitive detection of low-abundance analytes.^111^ Wang *et al.* developed a nano-hybrid microchip integrated with SERS to detect four melanoma-related protein biomarkers directly from 5 *μ*l of serum, without the need for extensive EVs pre-enrichment.^138^ The system employs a sandwich immunoassay format and achieves a signal amplification of 3.7- to 4.2-fold, enhancing both sensitivity and specificity while streamlining the workflow. Despite these advantages, SERS-based detection remains sensitive to substrate variability and hotspot reproducibility, potentially affecting consistency. Additionally, the platform's current application is limited to protein biomarkers, with unproven adaptability to other EVs contents such as miRNAs or lipids, which may constrain its broader diagnostic utility. Han *et al.* developed a SERS-integrated microfluidic platform with side filtration to quantify EVs biomarkers, CD63, vimentin (VIM), and EpCAM, in plasma from osteosarcoma patients [Fig. 5(d)]. The system achieved a detection limit of 2 EVs/*μ*l using just 50 *μ*l of plasma and completed analysis within 5 h, demonstrating strong diagnostic performance (100% sensitivity, 90% specificity, and 95% accuracy).^139^ These results highlight the platform's potential for accurate osteosarcoma classification and the utility of SERS in multiplexed EVs detection. Having said, that challenges remain for clinical translation. The reproducibility of Raman signal enhancement is limited by nanostructure uniformity and hotspot variability. Additionally, the high diagnostic accuracy was based on a small sample size and requires validation in broader patient populations. The use of multiple biomarkers also adds assay complexity, which may hinder standardization and scalability in clinical workflows. Gurudatt *et al.* developed an electrochemical microfluidic biochip that integrates a porous multi-orifice flow-fractionation (MOFF) channel with an indium-tin-oxide (ITO) sensor for rapid and sensitive detection of epithelial and mesenchymal markers on EVs, enabling evaluation of the epithelial–mesenchymal transition (EMT) index in pancreatic cancer.^141^ With a detection limit of 15 EVs/*μ*l and a clinical quantification threshold of 50 EVs/*μ*l, the platform delivers results within 5 min and effectively distinguishes IPMN (intraductal papillary mucinous neoplasm) from SCA samples, highlighting its diagnostic and commercialization potential. Yet, several limitations remain. EMT-based assessment, while biologically meaningful, can be affected by tumor heterogeneity and may not generalize across cancer types. The integration of MOFF and ITO components may complicate fabrication and hinder scalability. Moreover, the system's performance in complex clinical samples, particularly those with interfering biomolecules, requires further validation. These platforms often incorporate structurally complex components, such as dual filters or nanostructured substrates, which hinder scalability and increase production costs. Advancing clinical translation will require simplifying designs, improving manufacturability, and validating performance across diverse sample types.

##### b. EVs' RNA analysis.

In addition to the widespread utilization of proteins in the clinical evaluation of EVs, nucleic acids also play an indispensable role. The nucleic acids found within EVs, such as mRNA and miRNA, closely resemble the expression profiles of their originating cells, thereby providing valuable insights into the biological states and functions of those cells.^131,154,155^ Consequently, the transfer of nucleic acids between disparate cells via EVs' carriers is pivotal not only for intercellular communication but also significantly influences the onset and progression of various diseases.

Ramshani *et al.* developed a PCR- and chemical-free microfluidic platform integrating surface acoustic wave (SAW)-based lysis with an enrichment film sensor, enabling absolute quantification of EV-derived miRNAs (e.g., miR-21) in plasma or serum [Fig. 5(e)]. The system achieves a 1 pM detection limit from just 20 *μ*l of sample within 30 min, with <10% uncertainty, offering high analytical precision and streamlined operation.^24^ Even though this approach represents a major advance in EVs RNA analysis due to its integration and speed, several limitations persist. The efficacy of SAW-based lysis may vary with sample composition, affecting reproducibility, and its utility across broader miRNA panels or more complex fluids remains to be fully assessed. Compared to fluorescence or thermophoretic systems, this method reduces procedural complexity and cost but may require further calibration to ensure consistent lysis across diverse EVs subtypes. Subsequently, Sung *et al.* developed an integrated microfluidic platform that automates EVs separation, miRNA-21 extraction, reverse transcription (RT), and digital PCR (dPCR) for ovarian cancer (OvCa) diagnostics.^143^ The system operates without an external pump, streamlining workflow and enhancing suitability for point-of-care use. It achieves a detection limit of 11 copies/ml with quantification inaccuracy under 12%, outperforming conventional qPCR in sensitivity and precision. Despite its analytical strength, the platform has limitations. Its reliance on dPCR demands specialized, costly equipment, which may limit clinical scalability. Additionally, the integration of multiple processing steps could introduce variability, affecting reproducibility across diverse samples. Compared to PCR-free systems like SAW-based platforms, this method offers greater accuracy but with increased complexity and resource demands. Future research should focus on miniaturizing dPCR components, reducing processing time, and validating performance across larger, heterogeneous cohorts.

As EVs become increasingly important in clinical diagnostics, particularly for biomarker detection like miRNA and mRNA, efficient RNA analysis methods are essential.^156,157^ Microfluidic platforms offer significant advantages over traditional plate-based assays, including improved sensitivity, scalability, and operational efficiency.^158^ Unlike plate-based methods, which are limited by sample volume and long processing times, microfluidics enables rapid, high-throughput analysis with minimal sample waste, crucial in clinical settings. By integrating processes such as EVs separation, RNA extraction, and detection into a single, closed system, microfluidic platforms reduce contamination risks and human error.^159^ Their precise control over reaction conditions enhances RNA quantification accuracy, particularly for low-abundance biomarkers. Additionally, microfluidic devices are ideal for point-of-care applications, offering faster and more practical solutions than conventional laboratory assays. Overall, microfluidics provides superior sensitivity, automation, and efficiency, making it an excellent choice for scalable EVs RNA analysis in clinical diagnostics.

### B. Shared microfluidic platforms for EVs analysis

Distinct from the previously summarized integrated platforms for separation and detection, the platforms in question merge the enrichment and detection units into a singular chip structure, eschewing the need for additional detection areas.^130,160^ These platforms primarily incorporate finely designed microchannels for the capture of EVs, typically employing immunoaffinity capture methods on the inner surfaces of these channels, thus enhancing both the accuracy and efficiency of early clinical detection. To simultaneously achieve enrichment and detection without an independent detection area, these integrated platforms leverage the unique characteristics of various material interfaces, introducing diverse measurement technologies that facilitate separation and enrichment while enabling timely *in situ* detection to address varying experimental requirements.^161,162^ Table IV presents a summary of various shared microfluidic platforms.

**TABLE IV.** Comparison of shared microfluidic platforms for EVs.

Platform	Separation technique	Detection technique	Sample	Limit of detection	Work time	References
MoSERS microchip	MoS_2_ nanocavities	SERS	Blood	1.23%	30 min	163
TIRF	Anti-CD63 capture	TIRF microscope fluorescence detection	Plasma	NA	NA	164
PMMA and a nanoporous gold (Au) nanocluster	AuNC membrane capture	SPR	Urine	1000 particles/ml	NA	165
nPLEX	Nanopore capture	SPR	Ascites	3000 exosomes (670 aM)	NA	166
Exodisc	Filtration	ELISA	Urine	NA	1 h	104
ACE microarray chip	Microelectrode chip capture	Fluorescence detection	Blood	NA	90 min	10
DNA cage-based thermophoretic assay	DNA cage selective recognition	Thermoelectrophoresis	Serum	2.05 fM	NA	167
DTTA	RET-based DNA tetrahedron (FDT) label EVs	Thermoelectrophoresis	Serum	14 aM	NA	168
DNA three-way junction biosensor	Multicolor DNA biosensor label EVs	Fluorescence detection	Serum	0.116 g/ml	NA	169
Immunobiochip	Immunocapture	Fluorescence detection	Serum	NA	4 h	170

#### 1. Shared platforms of overall EVs levels analysis

To facilitate the comprehensive counting of EVs and real-time monitoring of total content on the same platform, researchers are dedicated to developing integrated analytical systems aimed at enhancing detection efficiency and ensuring data consistency.^171^ Such systems not only provide absolute counts of EVs but also allow for the real-time monitoring of overall EVs' content within samples, thereby offering more precise and comprehensive information to support clinical diagnostics and research. Current overall EVs analysis predominantly emphasizes the total count of patient-specific EVs, alongside the evaluation of therapeutic efficacy and strategies for disease prevention related to these individual EVs.^172^ However, this approach may overlook the intricate complexities of EVs' composition and their diverse functional roles in various biological contexts.^173^ A more refined strategy that incorporates both qualitative and quantitative assessments of EVs could yield deeper insights into their involvement in disease mechanisms and therapeutic responses, ultimately advancing personalized treatment approaches. Single EVs analysis relies on detection methods focused on individual EVs.^174^ Microfluidic technology is particularly well suited for this purpose due to its high efficiency, accuracy, and minimal sample requirements.^123^

**FIG. 6.**
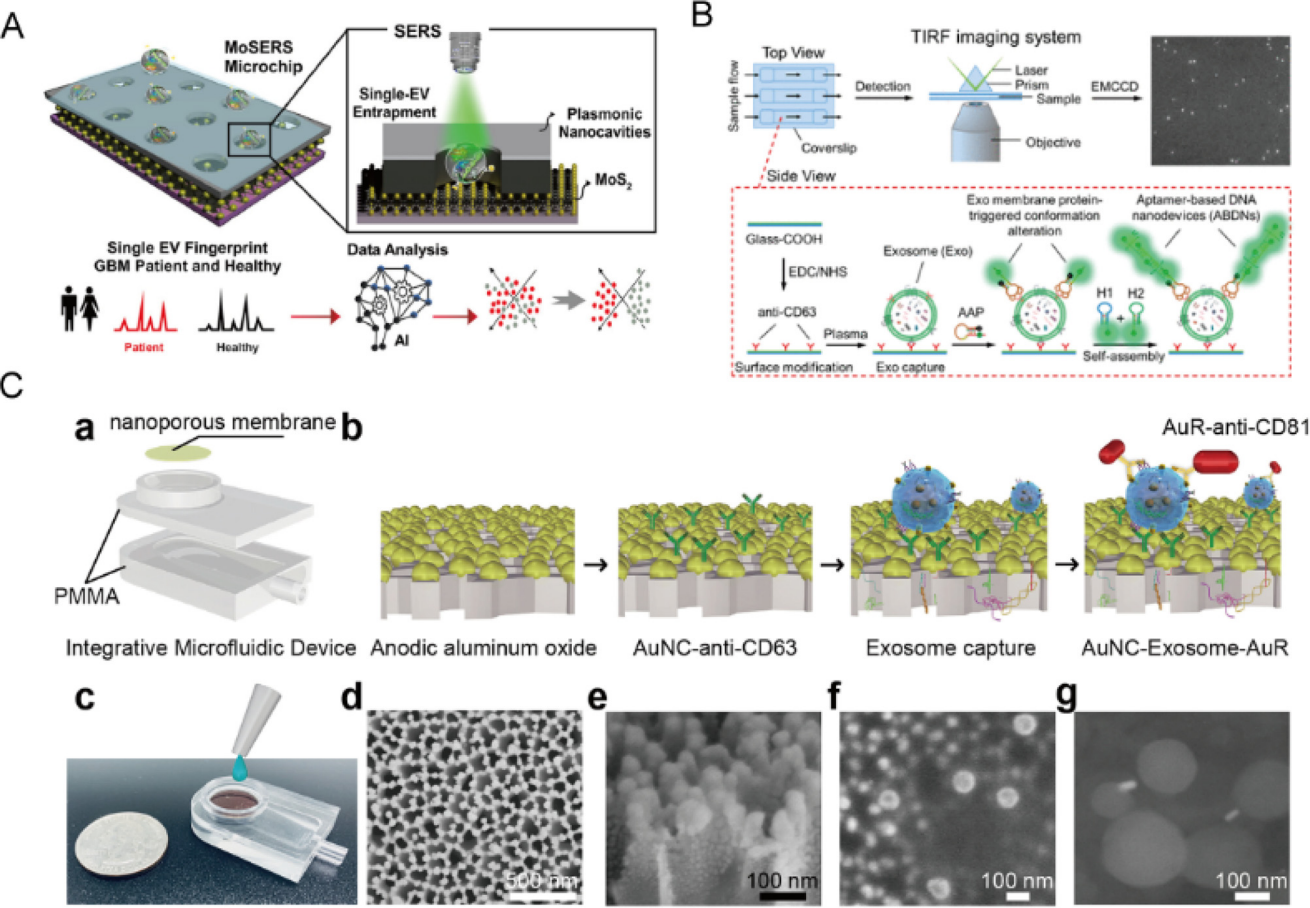
The shared platform was used for overall EVs content analysis. (a) The multi-channel fluid device combines embedded array nanocavity microchips (MoSERS microchips) with an SERS detection platform for analysis. Reprinted with permission from Jalali *et al.*, ACS Nano **17**(13), 12052–12071 (2023). Copyright 2023 Authors, licensed under a Creative Commons Attribution License.^163^ (b) A single-vesicle detection imaging platform based on total internal reflection fluorescence (TIRF) microscopy for the direct observation of tumor-derived EVs. Reprinted with permission from He *et al.*, Anal. Chem. **91**(4), 2768–2775 (2019). Copyright 2019 Authors, licensed under a Creative Commons Attribution License.^164^ (c) An integrated microfluidic apparatus for separation and *in situ* detection of polymethyl methacrylate (PMMA) and nanoporous gold (Au) nanocluster films modified with trapping antibodies was constructed. Reprinted with permission from Yang *et al.*, Biosens. Bioelectron. **163**, 112290 (2020). Copyright 2020 Elsevier.^165^

To enhance the sensitivity and efficiency of single-EV detection, Jalali *et al.* developed a multi-channel microfluidic device embedded with MoSERS nanocavity microchips [Fig. 6(a)]. These chips, composed of silver, zinc oxide, and molybdenum disulfide (MoS_2_), enable precise EVs separation and SERS-based detection in small sample volumes (<10 *μ*l).^163^ The system achieved 87% diagnostic accuracy and identified glioblastoma (GBM) mutations in blood samples from 12 patients, with sample preparation completed in just 30 min. Though the platform demonstrates strong potential for clinical diagnostics, especially in oncology, its complexity and reliance on nanomaterial fabrication may challenge scalability and routine clinical adoption. Although the MoSERS platform shows strong potential for single-EV diagnostics, particularly in GBM detection, challenges remain in reproducibility, fabrication complexity, and clinical integration. Future work should aim to improve material consistency, streamline device manufacturing, and validate performance across varied patient groups and EVs subtypes. He *et al.* developed a single-vesicle imaging platform using total internal reflection fluorescence (TIRF) microscopy for direct, quantitative analysis of tumor-derived EVs from as little as 1 *μ*l of plasma [Fig. 6(b)].^164^ The system captures EVs via antibodies bound to activated aptamer probes, triggering a hybridization chain reaction (HCR) targeting PTK7-exon and amplifying fluorescence signals for single-EV detection. While the platform offers high spatial resolution and sensitivity, its clinical applicability is limited by the need for specialized TIRF equipment and reliance on specific gene targets, which may reduce its generalizability across cancer types. Compared to other single-EV approaches such as MoSERS or electrochemical systems, this method excels in imaging precision but falls short in throughput and scalability. Future improvements should focus on simplifying system integration, broadening biomarker compatibility, and enhancing usability in clinical settings. Yang *et al.* developed an integrated microfluidic device combining polymethyl methacrylate (PMMA) with nanoporous gold (Au) nanocluster films for EVs separation and *in situ* detection from patient urine samples [Fig. 6(c)]. EVs are first captured on the AuNC membrane via antibodies and then hybridized with gold nanorods (AuRs), forming a sandwich complex that amplifies plasmonic scattering signals. This system achieved a detection limit below 1000 particles/ml, demonstrating strong potential for noninvasive cancer diagnostics.^165^ Despite its sensitivity and compact integration, the device faces challenges related to the fabrication of nanostructured gold surfaces and the complexity of dual-antibody binding, which may affect reproducibility in heterogeneous fluids. Compared to TIRF-based or electrochemical methods, this plasmonic approach offers enhanced signal strength but may sacrifice scalability and operational simplicity. Future efforts should aim to simplify fabrication, improve reproducibility, and ensure compatibility with point-of-care applications, potentially through automation or AI-assisted analysis.

#### 2. Shared platforms of overall EVs bioactive substances analysis

Recently, the advent of integrated analytical systems leveraging microfluidic platforms and nanotechnology has significantly enhanced the efficiency and accuracy of EVs' protein detection, making these systems particularly well suited for high-throughput screening of small-volume samples.^175^ Microfluidic EVs protein detection predominantly employs immunocapture methodologies. This approach involves the selective capture of target proteins via antibody-functionalized microchannels or microbeads, facilitating a streamlined “one-stop” operation encompassing EVs capture, separation, and protein detection.^176^

##### a. EVs' protein analysis.

**FIG. 7.**
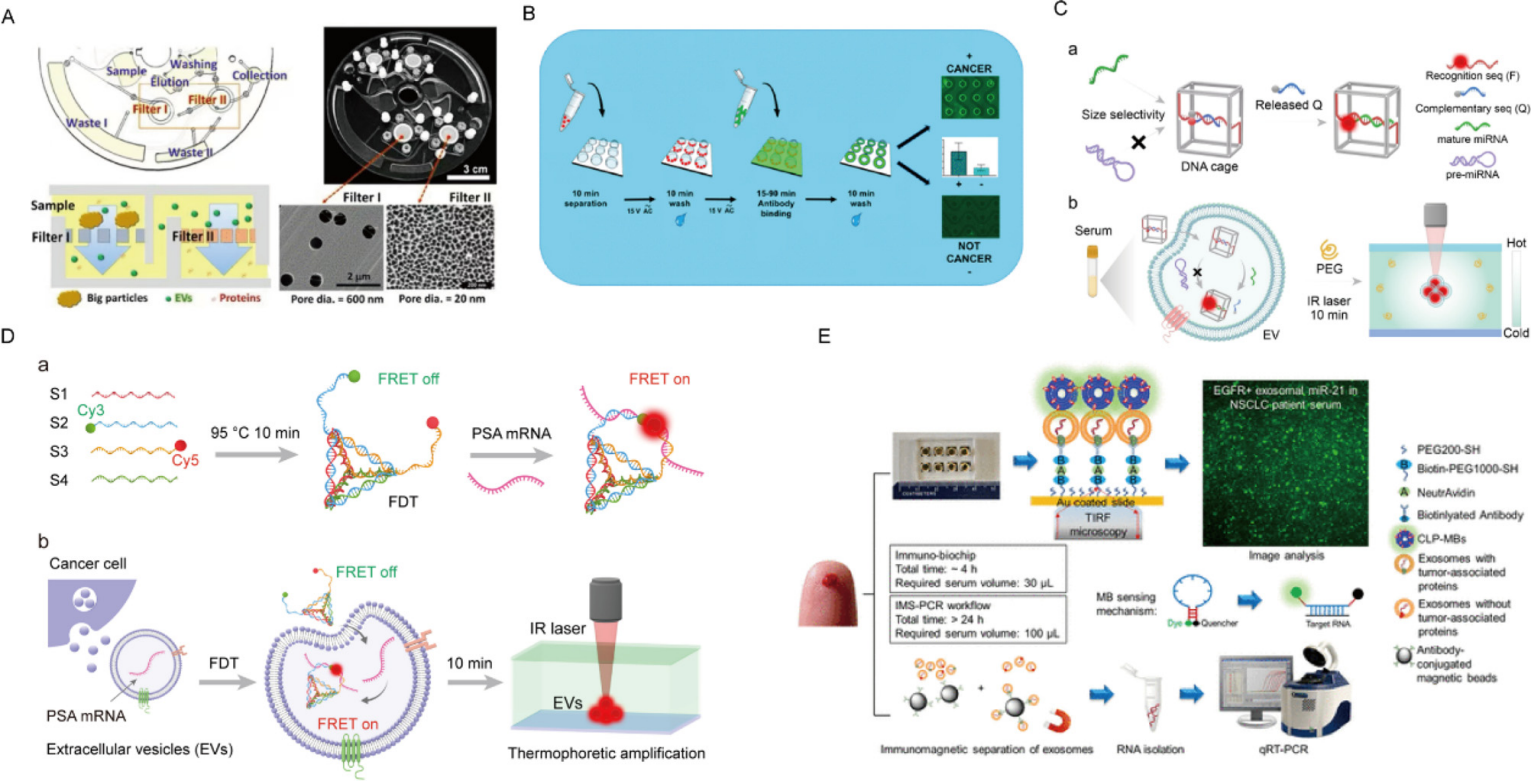
The shared platform was used for EVs bioactive substances analysis. (a) Exodisc microfluidic platform for urinary EVs enrichment and separation in patients with bladder cancer. Reprinted with permission from Woo *et al.*, ACS Nano **11**(2), 1360–1370 (2017). Copyright 2017 Authors, licensed under a Creative Commons Attribution license.^104^ (b) The device based on an alternating current electric (ACE) microarray chip directly separates and detects EVs in the whole blood of patients with pancreatic and colon cancer. Reprinted with permission from Lewis *et al.*, ACS Nano **12**(4), 3311–3320 (2018). Copyright 2018 American Chemical Society.^10^ (c) A DNA cage-based thermophoretic method enables highly sensitive *in situ* detection of microRNA within EVs. Reprinted with permission from Zhao *et al.*, Angew. Chem., Int. Ed. **62**(24), e202303121 (2023). Copyright 2023 Wiley VCH GmbH.^167^ (d) The DNA Tetrahedron-Based Thermostrophy Assay platform enables *in situ* detection of EVs mRNA, facilitating the distinction between prostate cancer (PCa) patients and those with benign prostatic hyperplasia (BPH). Reprinted with permission from Han *et al.*, Nano Today **38**, 101203 (2021). Copyright 2021 Elsevier.^168^ (e) An immunobiochemical chip that selectively captures tumor-associated proteins (such as EGFR and PD-L1) using antibodies and visualizes the fluorescence intensity of magnetic beads (MB) with total internal reflection fluorescence (TIRF) microscopy. Reprinted with permission from Yang *et al.*, ACS Appl. Mater. Interfaces **10**(50), 43375–43386 (2018). Copyright 2018 Authors, licensed under a Creative Commons Attribution License.^170^

To bolster the accuracy and repeatability of data derived from EVs protein analysis, Lee *et al.* introduced the nPLEX sensor, utilizing transmissive-based surface plasmon resonance (TSI) to enable sensitive, label-free EVs protein analysis through nanopore arrays. This approach improves accuracy and repeatability by tuning the cyclicality of nanopores and electromagnetic fields to detect distinct EVs proteins. While highly sensitive, its reliance on nanopore fine-tuning may limit scalability and application in complex biological matrices.^166^ In a departure from traditional assays like ELISA, Woo *et al.* presented the Exodisc microfluidic platform, integrating two nanofilter sets for high-sensitivity EVs protein analysis in bladder cancer urine samples [Fig. 7(a)]. With an efficient 30-min separation process, it demonstrates impressive 95% separation efficiency and a 100-fold concentration of target proteins like GAPDH and CD9.^104^ This platform stands out for its automation and potential in liquid biopsy applications, but its clinical adoption may face challenges related to sample variability and reproducibility in different patient populations. To separate and enrich EVs from whole blood for subsequent detection, Lewis *et al.* developed an AC electromotor (ACE) microarray chip device for separating EVs from small blood samples in just 20 min [Fig. 7(b)].^10^ This system achieved high sensitivity (99%) and decent specificity (82%) for biomarkers like glypican-1 and CD63. Nonetheless, it struggles with distinguishing EVs from other biomarkers, a limitation that may impact its clinical precision. The device exhibits limitations in its inability to effectively distinguish EVs from other high-content biomarkers, although it still facilitates a rapid workflow from a single drop of whole blood to the final detection result. Overall, these advancements show promise for liquid biopsy technologies, yet improvements in scalability, reproducibility, and specificity will be critical for broader clinical application.

##### b. EVs' RNA analysis.

The shared microfluidic platform optimizes the operational workflow by effectively integrating the separation of EVs with the extraction and analysis of RNA, thereby significantly enhancing both the efficiency and accuracy of the analytical process. In contrast to traditional methodologies, the design of this shared integrated platform not only mitigates sample loss at each procedural step but also enhances the overall repeatability of the experiments through automated processing.

The identification of EVs' RNA mainly focuses on miRNAs and mRNA. Zhao *et al.* used molecular probes to detect miRNAs in EVs, advancing their role in cancer diagnostics [Fig. 7(c)].^167^ The integrated platform for mRNA analysis minimizes losses and reduces processing time. While this approach shows promise for early cancer diagnostics, its effectiveness depends on the specificity and sensitivity of the probes, which can vary across different miRNA targets and biological samples. Han *et al.* developed a DNA tetrahedron-based thermophoretic assay (DTTA) for *in situ* mRNA detection within EVs, achieving a detection limit of 14 aM [Fig. 7(d)].^168^ The platform combines FDT with thermal accumulation to differentiate between prostate cancer (PCa) and benign prostatic hyperplasia (BPH) based on PSA (prostate-specific antigen) mRNA expression in EVs. While it efficiently integrates EVs separation, FDT internalization, and signal assessment in a single microfluidic device, the reliance on FRET (förster resonance energy transfer) and thermophoretic techniques may pose challenges in scalability and reproducibility, limiting its broader clinical adoption. Wang *et al.* developed an integrated microdevice using a DNA three-way junction for *in situ* detection of multiple miRNAs in breast cancer patients. The device detects miR-21, miR-27a, and miR-375 with detection limits of 0.116, 0.125, and 0.287 *μ*g/ml, respectively.^169^ By enabling simultaneous quantification of miRNAs without the need for EVs cleavage or complex RNA processing, the device offers a streamlined approach. However, its sensitivity may be limited by hybridization specificity and interference from abundant non-target RNAs in biological samples. Yang *et al.* developed an immunobiochip for *in situ* detection of EVs' miRNAs using total internal reflection fluorescence (TIRF) microscopy.^170^ The platform captures tumor-associated proteins (e.g., EGFR and PD-L1) and detects RNA via electrostatic interactions between a cationic lipid complex and EVs. This interaction activates molecular beacons (MB), allowing TIRF to measure RNA levels. While TIRF offers high sensitivity, its reliance on specialized equipment and challenges with complex biological matrices limit its practicality. While these platforms mark significant progress in EV-based RNA detection, their clinical application is limited by scalability, sensitivity, and methodological complexity. Future improvements should focus on simplifying the platforms, enhancing reproducibility, and optimizing performance in complex biological fluids. Additionally, integrating emerging technologies, like AI-driven data analysis, could address current analytical bottlenecks.

### C. Challenges for clinical adoption

Despite the significant promise of combined and shared microfluidic platforms for EVs analysis, several challenges must be overcome to facilitate their clinical adoption. The combined microfluidic platform presents complexities in design due to its reliance on multiple interconnected units for separation, enrichment, and detection. This serial configuration can complicate system integration and increase costs, which may limit its scalability and practicality in clinical environments. Furthermore, ensuring consistent and reliable performance across all interconnected units, especially when processing diverse clinical samples, remains a substantial challenge. While the platform's flexibility allows for a broad range of analytical tasks, this versatility often comes at the cost of extended processing times, which could hinder its suitability for real-time, point-of-care applications. In contrast, the shared microfluidic platform offers distinct advantages, such as compactness and the ability to perform real-time, *in situ* analysis. Yet, it faces limitations in accommodating diverse sample types and handling complex workflows. The integration of both enrichment and detection steps within a single unit can compromise sensitivity, particularly when analyzing low-abundance biomarkers.^160^ Additionally, the need to optimize both processes simultaneously can introduce potential interference, negatively affecting the quality of detection. This highlights the trade-off between convenience and performance, which must be carefully managed to ensure clinical reliability.^177^

From a clinical perspective, both platforms must meet stringent requirements for accuracy, reproducibility, and user accessibility. To gain acceptance, these microfluidic systems must demonstrate sensitivity and specificity comparable to existing gold-standard methods, ensuring their reliability in real-world diagnostic settings.^132^ Moreover, challenges related to regulatory approvals, cost-effectiveness, and the ability to function effectively in point-of-care settings are critical considerations for their widespread adoption. Addressing these technical, logistical, and practical barriers is essential for unlocking the full potential of microfluidic technologies in clinical diagnostics.

## IV. CONCLUSIONS

EVs have gained significant attention. These technologies offer notable advantages, including high automation, minimal sample volume requirements, and rapid processing speeds, making them highly promising for early diagnosis and disease monitoring. When compared to traditional methods, microfluidic platforms demonstrate superior sensitivity and lower detection limits. Nonetheless, despite substantial progress, several challenges and limitations continue to hinder their clinical translation. One of the primary barriers to widespread adoption is the issue of standardization. Currently, the design and operational conditions of microfluidic platforms often depend on specific laboratory settings, resulting in poor reproducibility across different laboratories. Furthermore, while microfluidic technologies excel in small sample volumes, scalability remains a challenge, particularly when applied to large-scale, complex biological samples. Sensitivity at low EVs concentrations also needs further improvement, which directly impacts their effectiveness in early-stage disease detection. Finally, regulatory hurdles impede the clinical application of these platforms, as the lack of standardized protocols and certification processes restricts their broader adoption. To overcome these limitations, future research should prioritize the standardization and simplification of microfluidic platforms. Developing more versatile and user-friendly chips will improve both reproducibility and scalability. Additionally, enhancing sensitivity for detecting low-concentration EVs and improving the handling of complex biological samples will be key to advancing their practical application. Additionally, leveraging emerging technologies, particularly AI-driven analytical methods, can accelerate data processing and interpretation, helping to resolve current analytical bottlenecks. By addressing these challenges, integrated microfluidic technologies have the potential to evolve into more precise and efficient tools for clinical diagnostics.

## Data Availability

The data that support the findings of this study are available from the corresponding authors upon reasonable request.

## References

[1] J. L. A. N. Murk, B. M. Humbel, U. Ziese, J. M. Griffith, G. Posthuma, J. W. Slot, A. J. Koster, A. J. Verkleij, H. J. Geuze, and M. J. Kleijmeer, “Endosomal compartmentalization in three dimensions: Implications for membrane fusion,” Proc. Natl. Acad. Sci. U. S. A. 100(23), 13332–13337 (2003). 10.1073/pnas.2232379100 14597718 PMC263806

[2] Y. Zhang, W. Wu, X. Pan, Y. Wang, C. Wu, L. Lu, X.-Y. Yu, and Y. Li, “Extracellular vesicles as novel therapeutic targets and diagnosis markers,” Extracell. Vesicle 1, 100017 (2022). 10.1016/j.vesic.2022.100017

[3] V. Vrablova, N. Kosutova, A. Blsakova, A. Bertokova, P. Kasak, T. Bertok, and J. Tkac, “Glycosylation in extracellular vesicles: Isolation, characterization, composition, analysis and clinical applications,” Biotechnol. Adv. 67, 108196 (2023). 10.1016/j.biotechadv.2023.108196 37307942

[4] S. Gurung, D. Perocheau, L. Touramanidou, and J. Baruteau, “The exosome journey: From biogenesis to uptake and intracellular signalling,” Cell Commun. Signal. 19(1), 1–19 (2021). 10.1186/s12964-021-00730-1 33892745 PMC8063428

[5] E. Eitan, C. Suire, S. Zhang, and M. P. Mattson, “Impact of lysosome status on extracellular vesicle content and release,” Ageing Res. Rev. 32, 65–74 (2016). 10.1016/j.arr.2016.05.001 27238186 PMC5154730

[6] D. K. Jeppesen, A. M. Fenix, J. L. Franklin, J. N. Higginbotham, Q. Zhang, L. J. Zimmerman, D. C. Liebler, J. Ping, Q. Liu, R. Evans, W. H. Fissell, J. G. Patton, L. H. Rome, D. T. Burnette, and R. J. Coffey, “Reassessment of exosome composition,” Cell 177(2), 428–445.e18 (2019). 10.1016/j.cell.2019.02.029 30951670 PMC6664447

[7] L. Ding, X. Yang, Z. Gao, C. Y. Effah, X. Zhang, Y. Wu, and L. Qu, “A holistic review of the state-of-the-art microfluidics for exosome separation: An overview of the current status, existing obstacles, and future outlook,” Small 17(29), 1–19 (2021). 10.1002/smll.202007174 34047052

[8] S. Cai, B. Luo, P. Jiang, X. Zhou, F. Lan, Q. Yi, and Y. Wu, “Immuno-modified superparamagnetic nanoparticles via host-guest interactions for high-purity capture and mild release of exosomes,” Nanoscale 10(29), 14280–14289 (2018). 10.1039/C8NR02871K 30014056

[9] R. Kalluri and K. M. McAndrews, “The role of extracellular vesicles in cancer,” Cell 186(8), 1610–1626 (2023). 10.1016/j.cell.2023.03.010 37059067 PMC10484374

[10] J. M. Lewis, A. D. Vyas, Y. Qiu, K. S. Messer, R. White, and M. J. Heller, “Integrated analysis of exosomal protein biomarkers on alternating current electrokinetic chips enables rapid detection of pancreatic cancer in patient blood,” ACS Nano 12(4), 3311–3320 (2018). 10.1021/acsnano.7b08199 29570265

[11] S. Keller, J. Ridinger, A. K. Rupp, J. W. G. Janssen, and P. Altevogt, “Body fluid derived exosomes as a novel template for clinical diagnostics,” J. Transl. Med. 9, 1–9 (2011). 10.1186/1479-5876-9-86 PMC3118335 21651777

[12] F. Momen-Heravi, L. Balaj, S. Alian, P. Y. Mantel, A. E. Halleck, A. J. Trachtenberg, C. E. Soria, S. Oquin, C. M. Bonebreak, E. Saracoglu, J. Skog, and W. P. Kuo, “Current methods for the isolation of extracellular vesicles,” Biol. Chem. 394(10), 1253–1262 (2013). 10.1515/hsz-2013-0141 23770532 PMC7075462

[13] S. Zhang, Y. Liu, X. Wang, L. Yang, H. Li, Y. Wang, M. Liu, X. Zhao, Y. Xie, Y. Yang, S. Zhang, Z. Fan, J. Dong, Z. Yuan, Z. Ding, Y. Zhang, and L. Hu, “SARS-CoV-2 binds platelet ACE2 to enhance thrombosis in COVID-19,” J. Hematol. Oncol. 13(1), 1–22 (2020). 10.1186/s13045-020-00954-7 32887634 PMC7471641

[14] V. Sokolova, A. K. Ludwig, S. Hornung, O. Rotan, P. A. Horn, M. Epple, and B. Giebel, “Characterisation of exosomes derived from human cells by nanoparticle tracking analysis and scanning electron microscopy,” Colloids Surf., B 87(1), 146–150 (2011). 10.1016/j.colsurfb.2011.05.013 21640565

[15] V. Sunkara, H. K. Woo, and Y. K. Cho, “Emerging techniques in the isolation and characterization of extracellular vesicles and their roles in cancer diagnostics and prognostics,” Analyst 141(2), 371–381 (2016). 10.1039/C5AN01775K 26535415

[16] J. Wang, X. Huang, J. Xie, Y. Han, Y. Huang, and H. Zhang, “Exosomal analysis: Advances in biosensor technology,” Clin. Chim. Acta 518, 142–150 (2021). 10.1016/j.cca.2021.03.026 33811925

[17] F. Yang, X. Liao, Y. Tian, and G. Li, “Exosome separation using microfluidic systems: Size-based, immunoaffinity-based and dynamic methodologies,” Biotechnol. J. 12(4), 1–9 (2017). 10.1002/biot.201600699 28166394

[18] S. Z. Shirejini and F. Inci, “The Yin and Yang of exosome isolation methods: Conventional practice, microfluidics, and commercial kits,” Biotechnol. Adv. 54, 107814 (2022). 10.1016/j.biotechadv.2021.107814 34389465

[19] K. A. Hyun, H. Gwak, J. Lee, B. Kwak, and H. I. Jung, “Salivary exosome and cell-free DNA for cancer detection,” Micromachines 9(7), 340 (2018). 10.3390/mi9070340 30424273 PMC6082266

[20] B. Mun, R. Kim, H. Jeong, B. Kang, J. Kim, H. Y. Son, J. Lim, H. W. Rho, E. K. Lim, and S. Haam, “An immuno-magnetophoresis-based microfluidic chip to isolate and detect HER2-Positive cancer-derived exosomes via multiple separation,” Biosens. Bioelectron. 239, 115592 (2023). 10.1016/j.bios.2023.115592 37603987

[21] D. Yang, W. Zhang, H. Zhang, F. Zhang, L. Chen, L. Ma, L. M. Larcher, S. Chen, N. Liu, Q. Zhao, P. H. L. Tran, C. Chen, R. N. Veedu, and T. Wang, “Progress, opportunity, and perspective on exosome isolation—Efforts for efficient exosome-based theranostics,” Theranostics 10(8), 3684–3707 (2020). 10.7150/thno.41580 32206116 PMC7069071

[22] P. Beekman, A. Enciso-Martinez, H. S. Rho, S. P. Pujari, A. Lenferink, H. Zuilhof, L. W. M. M. Terstappen, C. Otto, and S. Le Gac, “Immuno-capture of extracellular vesicles for individual multi-modal characterization using AFM, SEM and Raman spectroscopy,” Lab Chip 19(15), 2526–2536 (2019). 10.1039/C9LC00081J 31292600

[23] Z. Liao, Y. Zhang, Y. Li, Y. Miao, S. Gao, F. Lin, Y. Deng, and L. Geng, “Microfluidic chip coupled with optical biosensors for simultaneous detection of multiple analytes: A review,” Biosens. Bioelectron. 126, 697–706 (2019). 10.1016/j.bios.2018.11.032 30544083

[24] Z. Ramshani, C. Zhang, K. Richards, L. Chen, G. Xu, B. L. Stiles, R. Hill, S. Senapati, D. B. Go, and H. C. Chang, “Extracellular vesicle microRNA quantification from plasma using an integrated microfluidic device,” Commun. Biol. 2(1), 1–9 (2019). 10.1038/s42003-019-0435-1 31123713 PMC6527557

[25] K. Yi, Z. Zhang, P. Chen, X. Xi, X. Zhao, Y. Rong, F. Long, Q. Zhang, Y. Zhang, M. Gao, W. Liu, B. F. Liu, Z. Zhu, and F. Wang, “Tidal microfluidic chip-based isolation and transcriptomic profiling of plasma extracellular vesicles for clinical monitoring of high-risk patients with hepatocellular carcinoma-associated precursors,” Biosens. Bioelectron. 276, 117228 (2025). 10.1016/j.bios.2025.117228 39954520

[26] Y. K. Wang, Y. R. Bao, Y. X. Liang, Y. J. Chen, W. H. Huang, and M. Xie, “Current progress and prospect of microfluidic-based exosome investigation,” TrAC, Trends Anal. Chem. 168, 117310 (2023). 10.1016/j.trac.2023.117310

[27] T. Zhang, T. Zhou, Q. Cui, X. Feng, S. Feng, M. Li, Y. Yang, Y. Hosokawa, G. Tian, A. Q. Shen, and Y. Yalikun, “Active microfluidic platforms for particle separation and integrated sensing applications,” ACS Sensors. 10(8), 5299–5313 (2025). 10.1021/acssensors.5c01896 40720668

[28] D. Raju, S. Bathini, S. Badilescu, A. Ghosh, and M. Packirisamy, “Microfluidic platforms for the isolation and detection of exosomes: A brief review,” Micromachines 13(5), 730 (2022). 10.3390/mi13050730 35630197 PMC9147043

[29] Y. Zhou, H. Liu, and H. Chen, “Advancement in exosome isolation and label-free detection towards clinical diagnosis,” TrAC, Trends Anal. Chem. 179, 117874 (2024). 10.1016/j.trac.2024.117874

[30] G. M. Whitesides, “The origins and the future of microfluidics,” Nature 442(7101), 368–373 (2006). 10.1038/nature05058 16871203

[31] F. Arduini, S. Cinti, V. Scognamiglio, D. Moscone, and G. Palleschi, “How cutting-edge technologies impact the design of electrochemical (bio)sensors for environmental analysis. A review,” Anal. Chim. Acta 959, 15–42 (2017). 10.1016/j.aca.2016.12.035 28159104

[32] J. Chen, D. Chen, Y. Xie, T. Yuan, and X. Chen, “Progress of microfluidics for biology and medicine,” Nano-Micro Lett. 5(1), 66–80 (2013). 10.1007/BF03354852

[33] K. W. Witwer, E. I. Buzás, L. T. Bemis, A. Bora, C. Lässer, J. Lötvall, E. N. Nolte-'t Hoen, M. G. Piper, S. Sivaraman, J. Skog, C. Théry, M. H. Wauben, and F. Hochberg, “Standardization of sample collection, isolation and analysis methods in extracellular vesicle research,” J. Extracell. Vesicles 2(1), 1–25 (2013). 10.3402/jev.v2i0.20360 PMC3760646 24009894

[34] D. K. Jeppesen, M. L. Hvam, B. Primdahl-Bengtson, A. T. Boysen, B. Whitehead, L. Dyrskjøt, T. F. Ørntoft, K. A. Howard, and M. S. Ostenfeld, “Comparative analysis of discrete exosome fractions obtained by differential centrifugation,” J. Extracell. Vesicles 3(1), 1–16 (2014). 10.3402/jev.v3.25011 PMC4224706 25396408

[35] J. Nam, J. Yoon, H. Jee, W. S. Jang, and C. S. Lim, “High-throughput separation of microvesicles from whole blood components using viscoelastic fluid,” Adv. Mater. Technol. 5(12), 1–10 (2020). 10.1002/admt.202000612

[36] M. Wu, Y. Ouyang, Z. Wang, R. Zhang, P. H. Huang, C. Chen, H. Li, P. Li, D. Quinn, M. Dao, S. Suresh, Y. Sadovsky, and T. J. Huang, “Isolation of exosomes from whole blood by integrating acoustics and microfluidics,” Proc. Natl. Acad. Sci. U. S. A. 114(40), 10584–10589 (2017). 10.1073/pnas.1709210114 28923936 PMC5635903

[37] M. Tayebi, D. Yang, D. J. Collins, and Y. Ai, “Deterministic sorting of submicrometer particles and extracellular vesicles using a combined electric and acoustic field,” Nano Lett. 21(16), 6835–6842 (2021). 10.1021/acs.nanolett.1c01827 34355908

[38] M. Sancho-Albero, V. Sebastián, J. Sesé, R. Pazo-Cid, G. Mendoza, M. Arruebo, P. Martín-Duque, and J. Santamaría, “Isolation of exosomes from whole blood by a new microfluidic device: Proof of concept application in the diagnosis and monitoring of pancreatic cancer,” J. Nanobiotechnol. 18(1), 1–15 (2020). 10.1186/s12951-020-00701-7 PMC7579907 33092584

[39] H. M. Tay, S. Kharel, R. Dalan, Z. J. Chen, K. K. Tan, B. O. Boehm, S. C. J. Loo, and H. W. Hou, “Rapid purification of sub-micrometer particles for enhanced drug release and microvesicles isolation,” NPG Asia Mater. 9(9), e434 (2017). 10.1038/am.2017.175

[40] Y. Meng, Y. Zhang, M. Bühler, S. Wang, M. Asghari, A. Stürchler, B. Mateescu, T. Weiss, S. Stavrakis, and A. J. deMello, “Direct isolation of small extracellular vesicles from human blood using viscoelastic microfluidics,” Sci. Adv. 9(40), eadi5296 (2023). 10.1126/sciadv.adi5296 37801500 PMC10558121

[41] Y. S. Chen, C. Chen, C. P. K. Lai, and G. B. Lee, “Isolation and digital counting of extracellular vesicles from blood via membrane-integrated microfluidics,” Sens. Actuators, B 358, 131473 (2022). 10.1016/j.snb.2022.131473

[42] S. D. Ibsen, J. Wright, J. M. Lewis, S. Kim, S. Y. Ko, J. Ong, S. Manouchehri, A. Vyas, J. Akers, C. C. Chen, B. S. Carter, S. C. Esener, and M. J. Heller, “Rapid isolation and detection of exosomes and associated biomarkers from plasma,” ACS Nano 11(7), 6641–6651 (2017). 10.1021/acsnano.7b00549 28671449

[43] Q. Niu, Y. Shu, Y. Chen, Z. Huang, Z. Yao, X. Chen, F. Lin, J. Feng, C. Huang, H. Wang, H. Ding, C. Yang, and L. Wu, “A fluid multivalent magnetic interface for high-performance isolation and proteomic profiling of tumor-derived extracellular vesicles,” Angew. Chem., Int. Ed. 62(21), e202215337 (2023). 10.1002/anie.202215337 36959092

[44] J. Zhang, S. Yan, D. Yuan, G. Alici, N. T. Nguyen, M. Ebrahimi Warkiani, and W. Li, “Fundamentals and applications of inertial microfluidics: A review,” Lab Chip 16(1), 10–34 (2016). 10.1039/C5LC01159K 26584257

[45] E. K. Sackmann, A. L. Fulton, and D. J. Beebe, “The present and future role of microfluidics in biomedical research,” Nature 507(7491), 181–189 (2014). 10.1038/nature13118 24622198

[46] Z. Chen, L. Zhao, L. Wei, Z. Huang, P. Yin, X. Huang, H. Shi, B. Hu, and J. Tian, “River meander-inspired cross-section in 3D-printed helical microchannels for inertial focusing and enrichment,” Sens. Actuators, B 301, 127125 (2019). 10.1016/j.snb.2019.127125

[47] D. Huang, J. Man, D. Jiang, J. Zhao, and N. Xiang, “Inertial microfluidics: Recent advances,” Electrophoresis 41(24), 2166–2187 (2020). 10.1002/elps.202000134 33027533

[48] H. M. Tay, S. Y. Leong, X. Xu, F. Kong, M. Upadya, R. Dalan, C. Y. Tay, M. Dao, S. Suresh, and H. W. Hou, “Direct isolation of circulating extracellular vesicles from blood for vascular risk profiling in type 2 diabetes mellitus,” Lab Chip 21(13), 2511–2523 (2021). 10.1039/D1LC00333J 34042931

[49] S. Y. Leong, W. W. Lok, K. Y. Goh, H. B. Ong, H. M. Tay, C. Su, F. Kong, M. Upadya, W. Wang, E. Radnaa, R. Menon, M. Dao, R. Dalan, S. Suresh, D. W. T. Lim, and H. W. Hou, “High-throughput microfluidic extraction of platelet-free plasma for microRNA and extracellular vesicle analysis,” ACS Nano 18(8), 6623–6637 (2024). 10.1021/acsnano.3c12862 38348825

[50] K. Kang, S. S. Lee, K. Hyun, S. J. Lee, and J. M. Kim, “DNA-based highly tunable particle focuser,” Nat. Commun. 4, 2567 (2013). 10.1038/ncomms3567 24108276

[51] X. Lu and X. Xuan, “Continuous microfluidic particle separation via elasto-inertial pinched flow fractionation,” Anal. Chem. 87(12), 6389–6396 (2015). 10.1021/acs.analchem.5b01432 26005774

[52] C. Liu, J. Guo, F. Tian, N. Yang, F. Yan, Y. Ding, J. Wei, G. Hu, G. Nie, and J. Sun, “field-free isolation of exosomes from extracellular vesicles by microfluidic viscoelastic flows,” ACS Nano 11(7), 6968–6976 (2017). 10.1021/acsnano.7b02277 28679045

[53] C. Liu, B. Ding, C. Xue, Y. Tian, G. Hu, and J. Sun, “Sheathless focusing and separation of diverse nanoparticles in viscoelastic solutions with minimized shear thinning,” Anal. Chem. 88(24), 12547–12553 (2016). 10.1021/acs.analchem.6b04564 28193038

[54] G. D'Avino, F. Greco, and P. L. Maffettone, “Particle migration due to viscoelasticity of the suspending liquid and its relevance in microfluidic devices,” Annu. Rev. Fluid Mech. 49, 341–360 (2017). 10.1146/annurev-fluid-010816-060150

[55] J. Y. Kim, S. W. Ahn, S. S. Lee, and J. M. Kim, “Lateral migration and focusing of colloidal particles and DNA molecules under viscoelastic flow,” Lab Chip 12(16), 2807–2814 (2012). 10.1039/c2lc40147a 22776909

[56] D. D. Taylor and S. Shah, “Methods of isolating extracellular vesicles impact down-stream analyses of their cargoes,” Methods 87, 3–10 (2015). 10.1016/j.ymeth.2015.02.019 25766927

[57] A. Meggiolaro, V. Moccia, P. Brun, M. Pierno, G. Mistura, V. Zappulli, and D. Ferraro, “Microfluidic strategies for extracellular vesicle isolation: Towards clinical applications,” Biosensors 13(1), 50 (2022). 10.3390/bios13010050 36671885 PMC9855931

[58] D. Yu, Y. Li, M. Wang, J. Gu, W. Xu, H. Cai, X. Fang, and X. Zhang, “Exosomes as a new frontier of cancer liquid biopsy,” Mol. Cancer 21(1), 1–33 (2022). 10.1186/s12943-022-01509-9 35180868 PMC8855550

[59] G. Kim, M. C. Park, S. Jang, D. Han, H. Kim, W. Kim, H. Chun, and S. Kim, “Diffusion-based separation of extracellular vesicles by nanoporous membrane chip,” Biosensors 11(9), 347 (2021). 10.3390/bios11090347 34562937 PMC8472239

[60] A. N. Böing, E. van der Pol, A. E. Grootemaat, F. A. W. Coumans, A. Sturk, and R. Nieuwland, “Single-step isolation of extracellular vesicles by size-exclusion chromatography,” J. Extracell. Vesicles 3(1), 23430 (2014). 10.3402/jev.v3.23430 PMC4159761 25279113

[61] J. Z. Nordin, R. B. Bostancioglu, G. Corso, and S. El Andaloussi, “Tangential flow filtration with or without subsequent bind-elute size exclusion chromatography for purification of extracellular vesicles,” Methods Mol. Biol. 1953, 287–299 (2019). 10.1007/978-1-4939-9145-7_18 30912029

[62] Z. Li, C. Liu, Y. Cheng, Y. Li, J. Deng, L. Bai, L. Qin, H. Mei, M. Zeng, F. Tian, S. Zhang, and J. Sun, “Cascaded microfluidic circuits for pulsatile filtration of extracellular vesicles from whole blood for early cancer diagnosis,” Sci. Adv. 9(16), 1–15 (2023). 10.1126/sciadv.ade2819 PMC10121168 37083528

[63] P. Li and T. J. Huang, “Applications of acoustofluidics in bioanalytical chemistry,” Anal. Chem. 91(1), 757–767 (2019). 10.1021/acs.analchem.8b03786 30561981 PMC6493336

[64] P. Zhang, H. Bachman, A. Ozcelik, and T. J. Huang, “Acoustic microfluidics,” Annu. Rev. Anal. Chem. 13, 17–43 (2020). 10.1146/annurev-anchem-090919-102205 PMC7415005 32531185

[65] D. J. Collins, A. Neild, A. deMello, A. Q. Liu, and Y. Ai, “The Poisson distribution and beyond: Methods for microfluidic droplet production and single cell encapsulation,” Lab Chip 15(17), 3439–3459 (2015). 10.1039/C5LC00614G 26226550

[66] X. Ding, Z. Peng, S. C. S. Lin, M. Geri, S. Li, P. Li, Y. Chen, M. Dao, S. Suresh, and T. J. Huang, “Cell separation using tilted-angle standing surface acoustic waves,” Proc. Natl. Acad. Sci. U. S. A. 111(36), 12992–12997 (2014). 10.1073/pnas.1413325111 25157150 PMC4246961

[67] T. Laurell, F. Petersson, and A. Nilsson, “Chip integrated strategies for acoustic separation and manipulation of cells and particles,” Chem. Soc. Rev. 36(3), 492–506 (2007). 10.1039/B601326K 17325788

[68] K. Lee, H. Shao, R. Weissleder, and H. Lee, “Acoustic purification of extracellular microvesicles,” ACS Nano 9(3), 2321–2327 (2015). 10.1021/nn506538f 25672598 PMC4373978

[69] M. Wu, Z. Mao, K. Chen, H. Bachman, Y. Chen, J. Rufo, L. Ren, P. Li, L. Wang, and T. J. Huang, “Acoustic separation of nanoparticles in continuous flow,” Adv. Funct. Mater. 27(14), 1606039 (2017). 10.1002/adfm.201606039 29104525 PMC5668689

[70] C. Qian, H. Huang, L. Chen, X. Li, Z. Ge, T. Chen, Z. Yang, and L. Sun, “Dielectrophoresis for bioparticle manipulation,” Int. J. Mol. Sci. 15(10), 18281–18309 (2014). 10.3390/ijms151018281 25310652 PMC4227216

[71] Z. Yu, S. Lin, F. Xia, Y. Liu, D. Zhang, F. Wang, Y. Wang, Q. Li, J. Niu, C. Cao, D. Cui, N. Sheng, J. Ren, Z. Wang, and D. Chen, “ExoSD chips for high-purity immunomagnetic separation and high-sensitivity detection of gastric cancer cell-derived exosomes,” Biosens. Bioelectron. 194, 113594 (2021). 10.1016/j.bios.2021.113594 34474280

[72] S. Yan, Y. Liu, N. T. Nguyen, and J. Zhang, “Magnetophoresis-enhanced elasto-inertial migration of microparticles and cells in microfluidics,” Anal. Chem. 96(9), 3925–3932 (2024). 10.1021/acs.analchem.3c05803 38346322

[73] W. Olejarz, K. Sadowski, and K. Radoszkiewicz, “Extracellular vesicles in atherosclerosis: State of the art,” Int. J. Mol. Sci. 25(1), 388 (2024). 10.3390/ijms25010388 PMC10779125 38203558

[74] B. A. Cassiano, A. L. P. A. Silveira, Y. J. Kim, J. B. do Amaral, L. H. da Silva Nali, A. L. L. Bachi, L. D. Resende, F. A. H. Fonseca, M. C. de Oliveira Izar, I. D. Tuleta, J. R. Victor, D. Pallos, and C. N. França, “Role of circulating microparticles and cytokines in periodontitis associated with diabetes,” Front. Med. 11, 1–9 (2024). 10.3389/fmed.2024.1394300 PMC11381390 39253540

[75] J. Zhang, C. Chen, R. Becker, J. Rufo, S. Yang, J. Mai, P. Zhang, Y. Gu, Z. Wang, Z. Ma, J. Xia, N. Hao, Z. Tian, D. T. W. Wong, Y. Sadovsky, L. P. Lee, and T. J. Huang, “A solution to the biophysical fractionation of extracellular vesicles: Acoustic Nanoscale Separation via Wave-pillar Excitation Resonance (ANSWER),” Sci. Adv. 8(47), 1–10 (2022). 10.1126/sciadv.ade0640 PMC9683722 36417505

[76] C. H. Cheng, H. Yatsuda, S. H. Liu, W. N. Tsai, T. S. Cheng, S. Y. Chen, C. Y. F. Huang, H. C. Chang, and J. Kondoh, “An approach for measuring extracellular vesicle size using the attenuation-velocity change ratio of SH-SAW biosensors,” Anal. Chem. 97, 15234 (2025). 10.1021/acs.analchem.5c01881 40626837

[77] S. P. Zhang, J. Lata, C. Chen, J. Mai, F. Guo, Z. Tian, L. Ren, Z. Mao, P. H. Huang, P. Li, S. Yang, and T. J. Huang, “Digital acoustofluidics enables contactless and programmable liquid handling,” Nat. Commun. 9(1), 1–11 (2018). 10.1038/s41467-018-05297-z 30050088 PMC6062562

[78] Y. Wang, X. Tao, R. Tao, J. Zhou, Q. Zhang, D. Chen, H. Jin, S. Dong, J. Xie, and Y. Q. Fu, “Acoustofluidics along inclined surfaces based on AlN/Si Rayleigh surface acoustic waves,” Sens. Actuators, A 306, 111967 (2020). 10.1016/j.sna.2020.111967

[79] M. Singh, P. K. Tiwari, V. Kashyap, and S. Kumar, “Proteomics of extracellular vesicles: Recent updates, challenges and limitations,” Proteomes 13(1), 12–17 (2025). 10.3390/proteomes13010012 40137841 PMC11944546

[80] M. Sharma, M. Sheth, H. M. Poling, D. Kuhnell, S. M. Langevin, and L. Esfandiari, “Rapid purification and multiparametric characterization of circulating small extracellular vesicles utilizing a label-free lab-on-a-chip device,” Sci. Rep. 13(1), 1–12 (2023). 10.1038/s41598-023-45409-4 37880299 PMC10600140

[81] M. Tsamchoe, S. Petrillo, A. Lazaris, and P. Metrakos, “Isolation of extracellular vesicles from human plasma samples: The importance of controls,” Biotechnol. J. 18(6), 2200575 (2023). 10.1002/biot.202200575 36988156

[82] C. Ma, Z. Xu, K. Hao, L. Fan, W. Du, Z. Gao, C. Wang, Z. Zhang, N. Li, Q. Li, Q. Gao, and C. Yu, “Rapid isolation method for extracellular vesicles based on Fe_3_O_4_@ZrO_2_,” Front. Bioeng. Biotechnol. 12, 1–10 (2024). 10.3389/fbioe.2024.1399689 PMC11263208 39045537

[83] P. Li, M. Kaslan, S. H. Lee, J. Yao, and Z. Gao, “Progress in exosome isolation techniques,” Theranostics 7(3), 789–804 (2017). 10.7150/thno.18133 28255367 PMC5327650

[84] R. Tao, S. A. Hasan, H. Z. Wang, J. Zhou, J. T. Luo, G. McHale, D. Gibson, P. Canyelles-Pericas, M. D. Cooke, D. Wood, Y. Liu, Q. Wu, W. P. Ng, T. Franke, and Y. Q. Fu, “Bimorph material/structure designs for high sensitivity flexible surface acoustic wave temperature sensors,” Sci. Rep. 8(1), 9052 (2018). 10.1038/s41598-018-27324-1 29899347 PMC5998018

[85] J. Han, H. Hu, Y. Lei, Q. Huang, C. Fu, C. Gai, and J. Ning, “Optimization analysis of particle separation parameters for a standing surface acoustic wave acoustofluidic chip,” ACS Omega 8, 311 (2023). 10.1021/acsomega.2c04273 36643460 PMC9835635

[86] S. Li, L. Ren, P. H. Huang, X. Yao, R. A. Cuento, J. P. McCoy, C. E. Cameron, S. J. Levine, and T. J. Huang, “Acoustofluidic transfer of inflammatory cells from human sputum samples,” Anal. Chem. 88(11), 5655–5661 (2016). 10.1021/acs.analchem.5b03383 27183317 PMC5466821

[87] S. Lin, Z. Yu, D. Chen, Z. Wang, J. Miao, Q. Li, D. Zhang, J. Song, and D. Cui, “Progress in microfluidics-based exosome separation and detection technologies for diagnostic applications,” Small 16(9), 1903916 (2020). 10.1002/smll.201903916 31663295

[88] S. R. Vitale, J. A. Helmijr, M. Gerritsen, H. Coban, L. F. van Dessel, N. Beije, M. van der Vlugt-Daane, P. Vigneri, A. M. Sieuwerts, N. Dits, M. E. van Royen, G. Jenster, S. Sleijfer, M. Lolkema, J. W. M. Martens, and M. P. H. M. Jansen, “Detection of tumor-derived extracellular vesicles in plasma from patients with solid cancer,” BMC Cancer 21(1), 315 (2021). 10.1186/s12885-021-08007-z 33761899 PMC7992353

[89] D. M. Rissin, C. W. Kan, T. G. Campbell, S. C. Howes, D. R. Fournier, L. Song, T. Piech, P. P. Patel, L. Chang, A. J. Rivnak, E. P. Ferrell, J. D. Randall, G. K. Provuncher, D. R. Walt, and D. C. Duffy, “Single-molecule enzyme-linked immunosorbent assay detects serum proteins at subfemtomolar concentrations,” Nat. Biotechnol. 28(6), 595–599 (2010). 10.1038/nbt.1641 20495550 PMC2919230

[90] G. Bordanaba-Florit, F. Royo, S. G. Kruglik, and J. M. Falcón-Pérez, “Using single-vesicle technologies to unravel the heterogeneity of extracellular vesicles,” Nat. Protoc. 16(7), 3163–3185 (2021). 10.1038/s41596-021-00551-z 34135505

[91] A. C. Hatch, J. S. Fisher, A. R. Tovar, A. T. Hsieh, R. Lin, S. L. Pentoney, D. L. Yang, and A. P. Lee, “1-Million droplet array with wide-field fluorescence imaging for digital PCR,” Lab Chip 11(22), 3838–3845 (2011). 10.1039/c1lc20561g 21959960

[92] T. M. Blicharz, W. L. Siqueira, E. J. Helmerhorst, F. G. Oppenheim, P. J. Wexler, F. F. Little, and D. R. Walt, “Fiber-optic microsphere-based antibody array for the analysis of inflammatory cytokines in saliva,” Anal. Chem. 81(6), 2106–2114 (2009). 10.1021/ac802181j 19192965 PMC2765577

[93] N. Li, Y. Jiang, T. Lv, G. Li, and F. Yang, “Immunofluorescence analysis of breast cancer biomarkers using antibody-conjugated microbeads embedded in a microfluidic-based liquid biopsy chip,” Biosens. Bioelectron. 216, 114598 (2022). 10.1016/j.bios.2022.114598 36087400

[94] P. Li, J. Chen, Y. Chen, S. Song, X. Huang, Y. Yang, Y. Li, Y. Tong, Y. Xie, J. Li, S. Li, J. Wang, K. Qian, C. Wang, and L. Du, “Construction of exosome SORL1 detection platform based on 3D porous microfluidic chip and its application in early diagnosis of colorectal cancer,” Small 19(20), 2207381 (2023). 10.1002/smll.202207381 36799198

[95] C. Liu, X. Xu, B. Li, B. Situ, W. Pan, Y. Hu, T. An, S. Yao, and L. Zheng, “Single-exosome-counting immunoassays for cancer diagnostics,” Nano Lett. 18(7), 4226–4232 (2018). 10.1021/acs.nanolett.8b01184 29888919

[96] P. Zhang, X. Zhou, M. He, Y. Shang, A. L. Tetlow, A. K. Godwin, and Y. Zeng, “Ultrasensitive detection of circulating exosomes with a 3D-nanopatterned microfluidic chip,” Nat. Biomed. Eng. 3(6), 438–451 (2019). 10.1038/s41551-019-0356-9 31123323 PMC6556143

[97] R. Chinnappan, Q. Ramadan, and M. Zourob, “An integrated lab-on-a-chip platform for pre-concentration and detection of colorectal cancer exosomes using anti-CD63 aptamer as a recognition element,” Biosens. Bioelectron. 220, 114856 (2023). 10.1016/j.bios.2022.114856 36395728

[98] Y. Yan, Y. Wu, C. Lu, Y. Wei, J. Wang, B. Weng, W. Y. Huang, J. L. Zhang, K. Yang, and K. Lu, “Electrostatic self-assembly of CdS quantum dots with Co_9_S_8_ hollow nanotubes for enhanced visible light photocatalytic H_2_ production,” Molecules 29(15), 3530 (2024). 10.3390/molecules29153530 39124934 PMC11314185

[99] B. Lyu, X. Bao, D. Gao, X. Guo, X. Lu, and J. Ma, “Highly stable CsSnCl_3_ quantum dots grown in an ionic liquid/gelatin composite system through an in situ method,” Inorg. Chem. 61(14), 5672–5682 (2022). 10.1021/acs.inorgchem.2c00716 35333522

[100] J. Sobhanan, J. V. Rival, A. Anas, E. Sidharth Shibu, Y. Takano, and V. Biju, “Luminescent quantum dots: Synthesis, optical properties, bioimaging and toxicity,” Adv. Drug Delivery Rev. 197, 114830 (2023). 10.1016/j.addr.2023.114830 37086917

[101] S. Zeng, D. Baillargeat, H. P. Ho, and K. T. Yong, “Nanomaterials enhanced surface plasmon resonance for biological and chemical sensing applications,” Chem. Soc. Rev. 43(10), 3426–3452 (2014). 10.1039/c3cs60479a 24549396

[102] C. Wang, C. H. Huang, Z. Gao, J. Shen, J. He, A. MacLachlan, C. Ma, Y. Chang, W. Yang, Y. Cai, Y. Lou, S. Dai, W. Chen, F. Li, and P. Chen, “Nanoplasmonic sandwich immunoassay for tumor-derived exosome detection and exosomal PD-L1 profiling,” ACS Sens. 6(9), 3308–3319 (2021). 10.1021/acssensors.1c01101 34494426 PMC9275046

[103] X. Luo, S. Yan, G. Chen, Y. Wang, X. Zhang, J. Lan, J. Chen, and X. Yao, “A cavity induced mode hybridization plasmonic sensor for portable detection of exosomes,” Biosens. Bioelectron. 261, 116492 (2024). 10.1016/j.bios.2024.116492 38870828

[104] H. K. Woo, V. Sunkara, J. Park, T. H. Kim, J. R. Han, C. J. Kim, H. I. Choi, Y. K. Kim, and Y. K. Cho, “Exodisc for rapid, size-selective, and efficient isolation and analysis of nanoscale extracellular vesicles from biological samples,” ACS Nano 11(2), 1360–1370 (2017). 10.1021/acsnano.6b06131 28068467

[105] Z. Chen, S. B. Cheng, P. Cao, Q. F. Qiu, Y. Chen, M. Xie, Y. Xu, and W. H. Huang, “Detection of exosomes by ZnO nanowires coated three-dimensional scaffold chip device,” Biosens. Bioelectron. 122, 211–216 (2018). 10.1016/j.bios.2018.09.033 30265971

[106] M. J. A. Shiddiky, R. Vaidyanathan, M. Naghibosadat, S. Rauf, D. Korbie, L. G. Carrascosa, and M. Trau, “Detecting exosomes specifically: A microfluidic approach based on alternating current electrohydrodynamic induced nanoshearing,” Anal. Chem. 86(22), 674–676 (2014). 10.1021/AC502082B 25324037

[107] B. Sharma, R. R. Frontiera, A. I. Henry, E. Ringe, and R. P. Van Duyne, “SERS: Materials, applications, and the future,” Mater. Today 15(1–2), 16–25 (2012). 10.1016/S1369-7021(12)70017-2

[108] S. Stremersch, M. Marro, B. E. Pinchasik, P. Baatsen, A. Hendrix, S. C. De Smedt, P. Loza-Alvarez, A. G. Skirtach, K. Raemdonck, and K. Braeckmans, “Identification of individual exosome-like vesicles by surface enhanced Raman spectroscopy,” Small 12(24), 3292–3301 (2016). 10.1002/smll.201600393 27171437

[109] Y. Dai, S. Bai, C. Hu, K. Chu, B. Shen, and Z. J. Smith, “Combined morpho-chemical profiling of individual extracellular vesicles and functional nanoparticles without labels,” Anal. Chem. 92(7), 5585–5594 (2020). 10.1021/acs.analchem.0c00607 32162516

[110] W. Zhang, L. Jiang, R. J. Diefenbach, D. H. Campbell, B. J. Walsh, N. H. Packer, and Y. Wang, “Enabling sensitive phenotypic profiling of cancer-derived small extracellular vesicles using surface-enhanced Raman spectroscopy nanotags,” ACS Sens. 5(3), 764–771 (2020). 10.1021/acssensors.9b02377 32134252

[111] C. Nie, I. Shaw, and C. Chen, “Application of microfluidic technology based on surface-enhanced Raman scattering in cancer biomarker detection: A review,” J. Pharm. Anal. 13(12), 1429–1451 (2023). 10.1016/j.jpha.2023.08.009 38223444 PMC10785256

[112] J. U. Lee, W. H. Kim, H. S. Lee, K. H. Park, and S. J. Sim, “Quantitative and specific detection of exosomal miRNAs for accurate diagnosis of breast cancer using a surface-enhanced Raman scattering sensor based on plasmonic head-flocked gold nanopillars,” Small 15(17), 1804968 (2019). 10.1002/smll.201804968 30828996

[113] L. Xu, R. C. Gimple, W. B. Lau, B. Lau, F. Fei, Q. Shen, X. Liao, Y. Li, W. Wang, Y. He, M. Feng, H. Bu, W. Wang, and S. Zhou, “The present and future of the mass spectrometry-based investigation of the exosome landscape,” Mass Spectrom. Rev. 39(5–6), 745–762 (2020). 10.1002/mas.21635 32469100

[114] Z. Han, C. Peng, J. Yi, D. Zhang, X. Xiang, X. Peng, B. Su, B. Liu, Y. Shen, and L. Qiao, “Highly efficient exosome purification from human plasma by tangential flow filtration based microfluidic chip,” Sens. Actuators, B 333, 129563 (2021). 10.1016/j.snb.2021.129563

[115] L. Shan, Y. Qiao, L. Ma, X. Zhang, C. Chen, X. Xu, D. Li, S. Qiu, X. Xue, Y. Yu, Y. Guo, K. Qian, and J. Wang, “AuNPs/CNC nanocomposite with a ‘dual dispersion’ effect for LDI-TOF MS analysis of intact proteins in NSCLC serum exosomes,” Adv. Sci. 11(12), 2307360 (2024). 10.1002/advs.202307360 PMC10966532 38224220

[116] A. B. Hashkavayi, B. S. Cha, E. S. Lee, and K. S. Park, “Dual rolling circle amplification-enabled ultrasensitive multiplex detection of exosome biomarkers using electrochemical aptasensors,” Anal. Chim. Acta 1205, 339762 (2022). 10.1016/j.aca.2022.339762 35414380

[117] S. Singh, A. Numan, and S. Cinti, “Electrochemical nano biosensors for the detection of extracellular vesicles exosomes: From the benchtop to everywhere?,” Biosens. Bioelectron. 216, 114635 (2022). 10.1016/j.bios.2022.114635 35988430

[118] B. Jiang, T. Zhang, S. Liu, Y. Sheng, and J. Hu, “Polydopamine-assisted aptamer-carrying tetrahedral DNA microelectrode sensor for ultrasensitive electrochemical detection of exosomes,” J. Nanobiotechnol. 22(1), 55 (2024). 10.1186/s12951-024-02318-6 PMC10854160 38331774

[119] J. Ko, Y. Wang, J. C. T. Carlson, A. Marquard, J. Gungabeesoon, A. Charest, D. Weitz, M. J. Pittet, and R. Weissleder, “Single extracellular vesicle protein analysis using immuno-droplet digital polymerase chain reaction amplification,” Adv. Biosyst. 4(12), 1900307 (2020). 10.1002/adbi.201900307 PMC8491538 33274611

[120] L. Pasini, M. Notarangelo, A. Vagheggini, M. A. Burgio, L. Crinò, E. Chiadini, A. I. Prochowski, A. Delmonte, P. Ulivi, and V. G. D'Agostino, “Unveiling mutational dynamics in non-small cell lung cancer patients by quantitative EGFR profiling in vesicular RNA,” Mol. Oncol. 15(9), 2423–2438 (2021). 10.1002/1878-0261.12976 33942501 PMC8410558

[121] J. Ko, Y. Wang, K. Sheng, D. A. Weitz, and R. Weissleder, “Sequencing-based protein analysis of single extracellular vesicles,” ACS Nano 15(3), 5631–5638 (2021). 10.1021/acsnano.1c00782 33687214 PMC8742254

[122] S. Yokota, H. Kuramochi, K. Okubo, A. Iwaya, S. Tsuchiya, and T. Ichiki, “Extracellular vesicles nanoarray technology: Immobilization of individual extracellular vesicles on nanopatterned polyethylene glycol-lipid conjugate brushes,” PLoS One 14(10), e0224091 (2019). 10.1371/journal.pone.0224091 31648253 PMC6812765

[123] R. Friedrich, S. Block, M. Alizadehheidari, S. Heider, J. Fritzsche, E. K. Esbjörner, F. Westerlund, and M. Bally, “A nano flow cytometer for single lipid vesicle analysis,” Lab Chip 17(5), 830–841 (2017). 10.1039/C6LC01302C 28128381

[124] E. Willms, C. Cabañas, I. Mäger, M. J. A. Wood, and P. Vader, “Extracellular vesicle heterogeneity: Subpopulations, isolation techniques, and diverse functions in cancer progression,” Front. Immunol. 9, 738 (2018). 10.3389/fimmu.2018.00738 29760691 PMC5936763

[125] Y. Yang, G. Shen, H. Wang, H. Li, T. Zhang, N. Tao, X. Ding, and H. Yu, “Interferometric plasmonic imaging and detection of single exosomes,” Proc. Natl. Acad. Sci. U. S. A. 115(41), 10275–10280 (2018). 10.1073/pnas.1804548115 30249664 PMC6187158

[126] G. Li, W. Tang, and F. Yang, “Cancer liquid biopsy using integrated microfluidic exosome analysis platforms,” Biotechnol. J. 15(5), 1900225 (2020). 10.1002/biot.201900225 32032977

[127] S. Surappa, P. Multani, U. Parlatan, P. D. Sinawang, J. Kaifi, D. Akin, and U. Demirci, “Integrated ‘lab-on-a-chip’ microfluidic systems for isolation, enrichment, and analysis of cancer biomarkers,” Lab Chip 23(13), 2942–2958 (2023). 10.1039/D2LC01076C 37314731 PMC10834032

[128] H. Xu and B. C. Ye, “Integrated microfluidic platforms for tumor-derived exosome analysis,” TrAC, Trends Anal. Chem. 158, 116860 (2023). 10.1016/j.trac.2022.116860

[129] M. Logozzi, D. Mizzoni, D. F. Angelini, R. Di Raimo, M. Falchi, L. Battistini, and S. Fais, “Microenvironmental pH and exosome levels interplay in human cancer cell lines of different histotypes,” Cancers 10(10), 370 (2018). 10.3390/cancers10100370 30301144 PMC6210604

[130] W. Zhao, L. Zhang, Y. Ye, Y. Li, X. Luan, J. Liu, J. Cheng, Y. Zhao, M. Li, and C. Huang, “Microsphere mediated exosome isolation and ultra-sensitive detection on a dielectrophoresis integrated microfluidic device,” Analyst 146(19), 5962–5972 (2021). 10.1039/D1AN01061A 34494041

[131] M. Dwivedi, D. Ghosh, A. Saha, S. Hasan, D. Jindal, H. Yadav, A. Yadava, and M. Dwivedi, “Biochemistry of exosomes and their theranostic potential in human diseases,” Life Sci. 315, 121369 (2023). 10.1016/j.lfs.2023.121369 36639052

[132] S. Zhou, T. Hu, F. Zhang, D. Tang, D. Li, J. Cao, W. Wei, Y. Wu, and S. Liu, “Integrated microfluidic device for accurate extracellular vesicle quantification and protein markers analysis directly from human whole blood,” Anal. Chem. 92(1), 1574–1581 (2020). 10.1021/acs.analchem.9b04852 31779307

[133] H. Xu, C. Liao, P. Zuo, Z. Liu, and B. C. Ye, “Magnetic-based microfluidic device for on-chip isolation and detection of tumor-derived exosomes,” Anal. Chem. 90(22), 13451–13458 (2018). 10.1021/acs.analchem.8b03272 30234974

[134] Y. Lu, L. Ye, X. Jian, D. Yang, H. Zhang, Z. Tong, Z. Wu, N. Shi, Y. Han, and H. Mao, “Integrated microfluidic system for isolating exosome and analyzing protein marker PD-L1,” Biosens. Bioelectron. 204, 113879 (2022). 10.1016/j.bios.2021.113879 35180692

[135] Y. Li, S. Zhang, C. Liu, J. Deng, F. Tian, Q. Feng, L. Qin, L. Bai, T. Fu, L. Zhang, Y. Wang, and J. Sun, “Thermophoretic glycan profiling of extracellular vesicles for triple-negative breast cancer management,” Nat. Commun. 15(1), 2292 (2024). 10.1038/s41467-024-46557-5 38480740 PMC10937950

[136] Y. Wang, W. Gao, M. Sun, B. Feng, H. Shen, J. Zhu, X. Chen, and S. Yu, “A filter-electrochemical microfluidic chip for multiple surface protein analysis of exosomes to detect and classify breast cancer,” Biosens. Bioelectron. 239, 115590 (2023). 10.1016/j.bios.2023.115590 37607449

[137] Y. H. Kwon, S. Park, H. Jiang, N. G. Gurudatt, K. Lee, H. Jeong, C. Nie, J. Shin, K. A. Hyun, and H. I. Jung, “High-resolution spiral microfluidic channel integrated electrochemical device for isolation and detection of extracellular vesicles without lipoprotein contamination,” Biosens. Bioelectron. 267, 116792 (2025). 10.1016/j.bios.2024.116792 39307033

[138] J. Wang, Y. C. Kao, Q. Zhou, A. Wuethrich, M. S. Stark, H. Schaider, H. P. Soyer, L. L. Lin, and M. Trau, “An integrated microfluidic-SERS platform enables sensitive phenotyping of serum extracellular vesicles in early stage melanomas,” Adv. Funct. Mater. 32(3), 2010296 (2022). 10.1002/adfm.202010296

[139] Z. Han, X. Peng, Y. Yang, J. Yi, D. Zhao, Q. Bao, S. Long, S. X. Yu, X. X. Xu, B. Liu, Y. J. Liu, Y. Shen, and L. Qiao, “Integrated microfluidic-SERS for exosome biomarker profiling and osteosarcoma diagnosis,” Biosens. Bioelectron. 217, 114709 (2022). 10.1016/j.bios.2022.114709 36115123

[140] J. Qiu, Q. Guo, Y. Chu, C. Wang, H. Xue, Y. Zhang, H. Liu, G. Li, and L. Han, “Efficient EVs separation and detection by an alumina-nanochannel-array-membrane integrated microfluidic chip and an antibody barcode biochip,” Anal. Chim. Acta 1304, 342576 (2024). 10.1016/j.aca.2024.342576 38637043

[141] N. G. Gurudatt, H. Gwak, K. A. Hyun, S. E. Jeong, K. Lee, S. Park, M. J. Chung, S. E. Kim, J. H. Jo, and H. I. Jung, “Electrochemical detection and analysis of tumor-derived extracellular vesicles to evaluate malignancy of pancreatic cystic neoplasm using integrated microfluidic device,” Biosens. Bioelectron. 226, 115124 (2023). 10.1016/j.bios.2023.115124 36758487

[142] X. Dong, J. Chi, L. Zheng, B. Ma, Z. Li, S. Wang, C. Zhao, and H. Liu, “Efficient isolation and sensitive quantification of extracellular vesicles based on an integrated ExoID-Chip using photonic crystals,” Lab Chip 19(17), 2897–2904 (2019). 10.1039/C9LC00445A 31363724

[143] C. Y. Sung, C. C. Huang, Y. S. Chen, K. F. Hsu, and G. B. Lee, “Isolation and quantification of extracellular vesicle-encapsulated microRNA on an integrated microfluidic platform,” Lab Chip 21(23), 4660–4671 (2021). 10.1039/D1LC00663K 34739016

[144] Q. Tai, H. Yu, M. Gao, and X. Zhang, “In situ capturing and counting device for the specific depletion and purification of cancer-derived exosomes,” Anal. Chem. 95(35), 13113–13122 (2023). 10.1021/acs.analchem.3c01670 37609888

[145] J. Kowal, M. Tkach, and C. Théry, “Biogenesis and secretion of exosomes,” Curr. Opin. Cell Biol. 29(1), 116–125 (2014). 10.1016/j.ceb.2014.05.004 24959705

[146] S. Gurunathan, M. H. Kang, M. Jeyaraj, and J. H. Kim, “Platinum nanoparticles enhance exosome release in human lung epithelial adenocarcinoma cancer cells (A549): Oxidative stress and the ceramide pathway are key players,” Int. J. Nanomed. 16, 515–538 (2021). 10.2147/IJN.S291138 PMC7837572 33519199

[147] N. Mukerjee, A. Bhattacharya, S. Maitra, M. Kaur, S. Ganesan, S. Mishra, A. Ashraf, M. Rizwan, K. K. Kesari, T. A. Tabish, and N. D. Thorat, “Exosome isolation and characterization for advanced diagnostic and therapeutic applications,” Mater. Today Bio 31, 101613 (2025). 10.1016/j.mtbio.2025.101613 PMC11950786 40161926

[148] Y. Zhang, X. Tong, L. Yang, R. Yin, Y. Li, D. Zeng, X. Wang, and K. Deng, “A herringbone mixer based microfluidic device HBEXO-chip for purifying tumor-derived exosomes and establishing miRNA signature in pancreatic cancer,” Sens. Actuators, B 332, 129511 (2021). 10.1016/j.snb.2021.129511

[149] J. Kowal, G. Arras, M. Colombo, M. Jouve, J. P. Morath, B. Primdal-Bengtson, F. Dingli, D. Loew, M. Tkach, and C. Théry, “Proteomic comparison defines novel markers to characterize heterogeneous populations of extracellular vesicle subtypes,” Proc. Natl. Acad. Sci. U. S. A. 113(8), E968–E977 (2016). 10.1073/pnas.1521230113 26858453 PMC4776515

[150] B. Mun, H. Jeong, R. Kim, B. Gu, J. Kim, H. Y. Son, H. W. Rho, E. K. Lim, and S. Haam, “3D-Nanostructured microfluidic device arranged in a herringbone pattern for the highly effective capture of HER2-Positive cancer-derived exosomes in urine,” Chem. Eng. J. 482, 148851 (2024). 10.1016/j.cej.2024.148851

[151] Q. Niu, J. Gao, K. Zhao, X. Chen, X. Lin, C. Huang, Y. An, X. Xiao, Q. Wu, L. Cui, P. Zhang, L. Wu, and C. Yang, “Fluid nanoporous microinterface enables multiscale-enhanced affinity interaction for tumor-derived extracellular vesicle detection,” Proc. Natl. Acad. Sci. U. S. A. 119(44), e2213236119 (2022). 10.1073/pnas.2213236119 36306324 PMC9636968

[152] M. I. Y. Elmallah, P. Ortega-Deballon, L. Hermite, J. P. Pais-De-Barros, J. Gobbo, and C. Garrido, “Lipidomic profiling of exosomes from colorectal cancer cells and patients reveals potential biomarkers,” Mol. Oncol. 16(14), 2710–2718 (2022). 10.1002/1878-0261.13223 35524452 PMC9298677

[153] L. Wang, A. Abdulla, A. Wang, A. R. Warden, K. Z. Ahmad, Y. Xin, and X. Ding, “Sickle-like inertial microfluidic system for online rare cell separation and tandem label-free quantitative proteomics (Orcs-Proteomics),” Anal. Chem. 94(15), 6026–6035 (2022). 10.1021/acs.analchem.2c00679 35380437

[154] J. G. Van den Boorn, J. Daßler, C. Coch, M. Schlee, and G. Hartmann, “Exosomes as nucleic acid nanocarriers,” Adv. Drug Delivery Rev. 65(3), 331–335 (2013). 10.1016/j.addr.2012.06.011 22750807

[155] W. Ke and K. A. Afonin, “Exosomes as natural delivery carriers for programmable therapeutic nucleic acid nanoparticles (NANPs),” Adv. Drug Delivery Rev. 176, 113835 (2021). 10.1016/j.addr.2021.113835 PMC8440450 34144087

[156] J. Chisholm, S. Haas-Neill, P. Margetts, and K. Al-Nedawi, “Characterization of proteins, mRNAs, and miRNAs of circulating extracellular vesicles from prostate cancer patients compared to healthy subjects,” Front. Oncol. 12, 1–12 (2022). 10.3389/fonc.2022.895555 PMC9776661 36568159

[157] J. Cheng, K. Zhang, C. Qu, J. Peng, and L. Yang, “Non-Coding RNAs Derived from Extracellular Vesicles Promote Pre-Metastatic Niche Formation and Tumor Distant Metastasis,” Cancers (Basel). 15(7), 1–18 (2023). 10.3390/cancers15072158 PMC10093357 37046819

[158] Z. Tong, D. Yang, C. Shen, C. Li, X. Xu, Q. Li, Z. Wu, H. Ma, F. Chen, and H. Mao, “Rapid automated extracellular vesicle isolation and miRNA preparation on a cost-effective digital microfluidic platform,” Anal. Chim. Acta 1296, 342337 (2024). 10.1016/j.aca.2024.342337 38401929

[159] Z. Tong, X. Xu, C. Shen, D. Yang, Y. Li, Q. Li, W. Yang, F. Xu, Z. Wu, L. Zhou, C. Zhan, and H. Mao, “All-in-one multiple extracellular vesicle miRNA detection on a miniaturized digital microfluidic workstation,” Biosens. Bioelectron. 270, 116976 (2025). 10.1016/j.bios.2024.116976 39591923

[160] Y. S. Chen, Y. D. Ma, C. Chen, S. C. Shiesh, and G. B. Lee, “An integrated microfluidic system for on-chip enrichment and quantification of circulating extracellular vesicles from whole blood,” Lab Chip 19(19), 3305–3315 (2019). 10.1039/C9LC00624A 31495861

[161] S. Mross, S. Pierrat, T. Zimmermann, and M. Kraft, “Microfluidic enzymatic biosensing systems: A review,” Biosens. Bioelectron. 70, 376–391 (2015). 10.1016/j.bios.2015.03.049 25841121

[162] J. Park, J. S. Park, C. H. Huang, A. Jo, K. Cook, R. Wang, H. Y. Lin, J. Van Deun, H. Li, J. Min, L. Wang, G. Yoon, B. S. Carter, L. Balaj, G. S. Choi, C. M. Castro, R. Weissleder, and H. Lee, “An integrated magneto-electrochemical device for the rapid profiling of tumour extracellular vesicles from blood plasma,” Nat. Biomed. Eng. 5(7), 678–689 (2021). 10.1038/s41551-021-00752-7 34183802 PMC8437135

[163] M. Jalali, C. del Real Mata, L. Montermini, O. Jeanne, I. I. Hosseini, Z. Gu, C. Spinelli, Y. Lu, N. Tawil, M. C. Guiot, Z. He, S. Wachsmann-Hogiu, R. Zhou, K. Petrecca, W. W. Reisner, J. Rak, and S. Mahshid, “MoS_2_-plasmonic nanocavities for Raman spectra of single extracellular vesicles reveal molecular progression in glioblastoma,” ACS Nano 17(13), 12052–12071 (2023). 10.1021/acsnano.2c09222 37366177 PMC10339787

[164] D. He, S. L. Ho, H. N. Chan, H. Wang, L. Hai, X. He, K. Wang, and H. W. Li, “Molecular-recognition-based DNA nanodevices for enhancing the direct visualization and quantification of single vesicles of tumor exosomes in plasma microsamples,” Anal. Chem. 91(4), 2768–2775 (2019). 10.1021/acs.analchem.8b04509 30644724

[165] Q. Yang, L. Cheng, L. Hu, D. Lou, T. Zhang, J. Li, Q. Zhu, and F. Liu, “An integrative microfluidic device for isolation and ultrasensitive detection of lung cancer-specific exosomes from patient urine,” Biosens. Bioelectron. 163, 112290 (2020). 10.1016/j.bios.2020.112290 32568696

[166] H. Im, H. Shao, Y. I. Park, V. M. Peterson, C. M. Castro, R. Weissleder, and H. Lee, “Label-free detection and molecular profiling of exosomes with a nano-plasmonic sensor,” Nat. Biotechnol. 32(5), 490–495 (2014). 10.1038/nbt.2886 24752081 PMC4356947

[167] S. Zhao, S. Zhang, H. Hu, Y. Cheng, K. Zou, J. Song, J. Deng, L. Li, X. B. Zhang, G. Ke, and J. Sun, “Selective in situ analysis of mature microRNAs in extracellular vesicles using a DNA cage-based thermophoretic assay,” Angew. Chem., Int. Ed. 62(24), e202303121 (2023). 10.1002/anie.202303121 37078239

[168] Z. Han, F. Wan, J. Deng, J. Zhao, Y. Li, Y. Yang, Q. Jiang, B. Ding, C. Liu, B. Dai, and J. Sun, “Ultrasensitive detection of mRNA in extracellular vesicles using DNA tetrahedron-based thermophoretic assay,” Nano Today 38, 101203 (2021). 10.1016/j.nantod.2021.101203

[169] H. Wang, D. He, K. Wan, X. Sheng, H. Cheng, J. Huang, X. Zhou, X. He, and K. Wang, “In situ multiplex detection of serum exosomal microRNAs using an all-in-one biosensor for breast cancer diagnosis,” Analyst 145(9), 3289–3296 (2020). 10.1039/D0AN00393J 32255115

[170] Y. Yang, E. Kannisto, G. Yu, M. E. Reid, S. K. Patnaik, and Y. Wu, “An immuno-biochip selectively captures tumor-derived exosomes and detects exosomal RNAs for cancer diagnosis,” ACS Appl. Mater. Interfaces 10(50), 43375–43386 (2018). 10.1021/acsami.8b13971 30451486 PMC8628516

[171] M. Shen, K. Di, H. He, Y. Xia, H. Xie, R. Huang, C. Liu, M. Yang, S. Zheng, N. He, and Z. Li, “Progress in exosome associated tumor markers and their detection methods,” Mol. Biomed. 1(1), 1–25 (2020). 10.1186/s43556-020-00002-3 35006428 PMC8603992

[172] M. Banijamali, P. Höjer, A. Nagy, P. Hååg, E. P. Gomero, C. Stiller, V. O. Kaminskyy, S. Ekman, R. Lewensohn, A. E. Karlström, K. Viktorsson, and A. Ahmadian, “Characterizing single extracellular vesicles by droplet barcode sequencing for protein analysis,” J. Extracell. Vesicles 11(11), e12277 (2022). 10.1002/jev2.12277 36329610 PMC9633998

[173] Z. Sun, X. Chen, R. Niu, H. Chen, Y. Zhu, C. Zhang, L. Wang, H. Mou, H. Zhang, and Y. Luo, “Liposome fusogenic enzyme-free circuit enables high-fidelity determination of single exosomal RNA,” Mater. Today Bio 19, 100613 (2023). 10.1016/j.mtbio.2023.100613 PMC10060373 37009069

[174] M. S. Panagopoulou, A. W. Wark, D. J. S. Birch, and C. D. Gregory, “Phenotypic analysis of extracellular vesicles: A review on the applications of fluorescence,” J. Extracell. Vesicles 9(1), 1710020 (2020). 10.1080/20013078.2019.1710020 32002172 PMC6968689

[175] K. Li, Z. P. Hong, Y. X. Li, Y. Li, and J. L. Yang, “Clinical significance of CD151 expression in non-small cell lung cancer,” Chin. J. Cancer Prev. Treat. 21(1), 34–38 (2014). 10.47056/0365-9615-2022-174-12-756-760

[176] S. Jeong, J. Park, D. Pathania, C. M. Castro, R. Weissleder, and H. Lee, “Integrated magneto-electrochemical sensor for exosome analysis,” ACS Nano 10(2), 1802–1809 (2016). 10.1021/acsnano.5b07584 26808216 PMC4802494

[177] S. Movahedi, F. Bahramian, M. Ahmadi, N. Pouyanfar, R. Masoudifar, M. Ghalkhani, C. M. Hussain, R. Keçili, S. Siavashy, and F. Ghorbani-Bidkorpeh, “Next-generation microfluidics based on artificial intelligence: Applications for food sample analysis,” Microchem. J. 212, 113395 (2025). 10.1016/j.microc.2025.113395

